# Identification of a hybrid myocardial zone in the mammalian heart after birth

**DOI:** 10.1038/s41467-017-00118-1

**Published:** 2017-07-20

**Authors:** Xueying Tian, Yan Li, Lingjuan He, Hui Zhang, Xiuzhen Huang, Qiaozhen Liu, Wenjuan Pu, Libo Zhang, Yi Li, Huan Zhao, Zhifu Wang, Jianhong Zhu, Yu Nie, Shengshou Hu, David Sedmera, Tao P. Zhong, Ying Yu, Li Zhang, Yan Yan, Zengyong Qiao, Qing-Dong Wang, Sean M. Wu, William T. Pu, Robert H. Anderson, Bin Zhou

**Affiliations:** 1 The State Key Laboratory of Cell Biology, CAS Center for Excellence in Molecular Cell Science, Shanghai Institute of Biochemistry and Cell Biology, Chinese Academy of Sciences, University of Chinese Academy of Sciences, Shanghai, 200031 China; 2 Key Laboratory of Nutrition and Metabolism, Institute for Nutritional Sciences, Shanghai Institutes for Biological Sciences, Chinese Academy of Sciences, University of Chinese Academy of Sciences, Shanghai, 200031 China; 3 School of Life Science and Technology, Shanghai Tech University, Shanghai, 201210 China; 4 Department of Neurosurgery, Huashan Hospital, State Key Laboratory of Medical Neurobiology, Institutes of Brain Science and Shanghai Medical College, Fudan University, Shanghai, 200032 China; 5 State Key Laboratory of Cardiovascular Disease, Fuwai Hospital, National Center for Cardiovascular Disease, Chinese Academy of Medical Sciences and Peking Union Medical College, Beijing, 100037 China; 6 Institute of Anatomy, First Faculty of Medicine, Charles University; Institute of Physiology The Czech Academy of Sciences, Prague, 12800 Czech Republic; 7 State Key Laboratory of Genetic Engineering, School of Life Sciences, Fudan University, Shanghai, 200433 China; 8 Department of Pharmacology, School of Basic Medical Sciences, Tianjin Medical University, Tianjin, 300070 China; 9 Department of Cardiology, The First Affiliated Hospital, School of Medicine, Zhejiang University, 79 Qingchun Road, Hangzhou, Zhejiang 310003 China; 10 Cardiology Department, Zhongshan Hospital, Fudan University, Shanghai, 200032 China; 11 Department of Cardiovascular Medicine, Southern Medical University Affiliated Fengxian Hospital, Shanghai, 201499 China; 12 Cardiovascular and Metabolic Diseases, Innovative Medicines and Early Clinical Development Biotech Unit, AstraZeneca, Mölndal, 43183 Sweden; 13 Division of Cardiovascular Medicine, Department of Medicine, Stanford Cardiovascular Institute, Stanford University School of Medicine, Stanford, Caliornia 94305 USA; 14 Harvard Stem Cell Institute, Harvard University, Cambridge, Massachusetts 02138 USA; 15 Department of Cardiology, Children’s Hospital Boston, 300 Longwood Avenue, Boston, Massachusetts 02115 USA; 16 Institute of Genetic Medicine, Newcastle University, Newcastle-upon-Tyne, NE1 7RU UK; 17 Key Laboratory of Regenerative Medicine of Ministry of Education, Institute of Aging and Regenerative Medicine, Jinan University, Guangzhou, 510632 China

## Abstract

Noncompaction cardiomyopathy is characterized by the presence of extensive trabeculations, which could lead to heart failure and malignant arrhythmias. How trabeculations resolve to form compact myocardium is poorly understood. Elucidation of this process is critical to understanding the pathophysiology of noncompaction disease. Here we use genetic lineage tracing to mark the Nppa^+^ or Hey2^+^ cardiomyocytes as trabecular and compact components of the ventricular wall. We find that Nppa^+^ and Hey2^+^ cardiomyocytes, respectively, from the endocardial and epicardial zones of the ventricular wall postnatally. Interposed between these two postnatal layers is a hybrid zone, which is composed of cells derived from both the Nppa^+^ and Hey2^+^ populations. Inhibition of the fetal Hey2^+^ cell contribution to the hybrid zone results in persistence of excessive trabeculations in postnatal heart. Our findings indicate that the expansion of Hey2^+^ fetal compact component, and its contribution to the hybrid myocardial zone, are essential for normal formation of the ventricular walls.

## Abstract (web summary)

Fetal trabecular muscles in the heart undergo a poorly described morphogenetic process that results into a solidified compact myocardium after birth. Tian et al. show that cardiomyocytes in the fetal compact layer also contribute to this process, forming a hybrid myocardial zone that is composed of cells derived from both trabecular and compact layers.

## Introduction

During development of the heart, cardiomyocytes extend into the ventricular chamber lumen to form a meshwork of trabeculations^1–5^. During perinatal stages, this interwoven mesh of muscles undergoes a poorly described morphogenetic process that results in its disappearance, leaving behind the postnatal ventricular walls that are largely composed of compact myocardium, with relatively smooth endocardial surfaces^6^. It has often been presumed that perturbation of this morphogenic process produces so-called left ventricular noncompaction cardiomyopathy (LVNC), which typically is characterized by the persistence of an excessive lace-like network of fine trabeculations^7, 8^. The presence of deep intertrabecular recesses may lead to cardiac failure, malignant arrhythmias, and thromboembolism, with the latter process potentially causing stroke and coronary arterial occlusion^9–11^. LVNC is now considered as the third most common type of cardiomyopathy, affecting up to 0.3% of the general population^12, 13^. In this setting, the most luminal component of trabecular meshwork is usually relatively well compacted, and supports the papillary muscles, which develop by a process of compaction of pre-existing trabeculations^14^. The middle part of the wall, in contrast, retains a lace-like configuration with deep intertrabecular endocardial spaces^7, 8, 15^. Recent studies on LVNC patients, and also genetic mouse models, showed that thinning of the compact myocardium is often part of the phenotype^9, 16–19^, indicating a reduced proliferation of the cardiomyocytes in the compact layer is involved in the pathogenesis of LVNC.

Better understanding of the LVNC pathogenesis requires unraveling the developmental process by which the trabecular meshwork resolves, and how this resolution is related to the formation of the mature left ventricular wall. Two mechanisms for this morphogenic transformation, compact myocardial expansion and trabecular coalescence, have been hypothesized (Supplementary Fig. 1a)^20^. In the compact myocardial expansion model, it is presumed that the late embryonic or perinatal expansion of compact myocardium subsequently fills the spaces within the trabecular meshwork, and gradually shapes these trabeculations into the solidified compact myocardial wall of the postnatal heart. The trabecular coalescence model hypothesizes that the trabeculations expand and coalesce by themselves, without receiving any significant contribution from the initial fetal compact myocardium, thus producing in the solidified compact wall of the postnatal heart. Up untill now, there has been no direct evidence to distinguish these models.

Here, we used genetic lineage tracing to study the fate of the embryonic trabecular and compact myocardial components of the ventricular walls in the postnatal heart. The present study mainly aims to delineate the cellular process of trabecular compaction in the developing heart, which provides a basis for further understanding the pathophysiology of noncompaction.

## Results

### *Nppa* and *Hey2* are markers for trabecular and compact myocardium, respectively

**Fig. 1:**
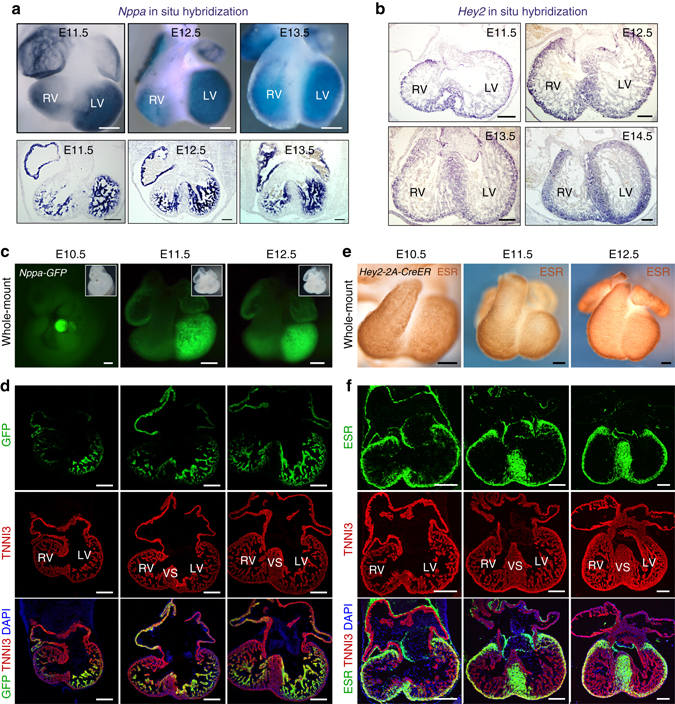
Nppa and Hey2 are expressed in trabecular and compact myocardium, respectively. **a** Whole-mount and sectional in situ hybridization of *Nppa* on E11.5–E13.5 hearts. **b** In situ hybridization of *Hey2* on E11.5–E14.5 heart sections. **c** Whole-mount fluorescence view of E10.5–E12.5 *Nppa-GFP* embryos or hearts. *Inserts* are bright-field view of embryos or hearts. **d** Immunostaining for GFP as surrogate for Nppa and cardiomyocyte marker TNNI3 on *Nppa-GFP* heart sections showed GFP is restricted to trabecular myocardium. **e** Whole-mount ESR staining of E10.5–E12.5 *Hey2-2A-CreER* mice hearts. **f** Immunostaining for ESR (as surrogate for Hey2) and TNNI3 on *Hey2-2A-CreER* heart sections showed ESR expression is enriched in compact myocardium but not in atrial or trabecular myocardium. Scale bars, 200 µm. Magnified images are shown in Supplementary Fig. 3b. Each image is a representative of four individual samples

We first used scanning electron microscopy to visualize the changes in myocardial architecture that occur during the embryonic and neonatal periods. The trabecular meshwork was readily detected from E10.5 to E14.5. The meshwork was less obvious in the later embryonic stages, and had almost disappeared by the neonatal period (Supplementary Fig. 1b). We next used *Nppa* and *Hey2* as specific molecular markers for the trabecular and compact layers of the ventricular myocardium, respectively^21^. In situ hybridization showed that *Nppa* was restricted to the trabeculations, whereas *Hey2* was enriched in the compact layer (Fig. 1a, b). We generated an *Nppa-GFP* knock-in mouse line (Supplementary Fig. 2a), and showed that green fluorescent protein (GFP), as a surrogate for endogenous *Nppa* expression, was largely restricted to trabecular cardiomyocytes at E11.5 and E12.5 (Fig. 1c, d and Supplementary Fig. 2b). The larger part of the left ventricle (LV) trabecular myocardium expressed GFP, whereas expression in the right ventricle (RV) trabecular myocardium was less extensive (Fig. 1d and Supplementary Fig. 2b). Using CRISPR/Cas9 technology, we generated a *Hey2-2A-CreER* knock-in allele, in which a Cre recombinase-estrogen hormone binding domain protein is controlled by *Hey2* regulatory elements (Supplementary Fig. 3a). Immunostaining for the estrogen receptor (ESR) component of CreER, as a surrogate for endogenous Hey2 expression, confirmed that the fusion protein CreER was expressed in the cardiomyocytes of the compact myocardium of both ventricles, whereas little expression was observed in the trabecular myocardium (Fig. 1e, f and Supplementary Fig. 3b). The fusion protein was also highly expressed throughout the forming interventricular septum at E11.5 and E12.5 (Fig. 1e, f and Supplementary Fig. 3b). Thus, by using *Nppa* and *Hey2*, we could distinguish between trabecular and compact ventricular myocardium in mid-embryonic stages (e.g., E12.5).

### Identification of a hybrid myocardial zone in postnatal heart

**Fig. 2:**
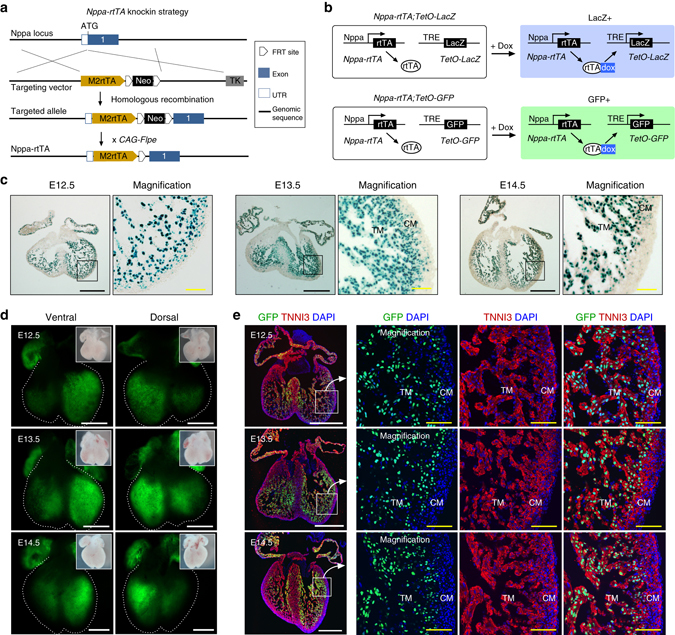
Generation and characterization of Nppa-rtTA mouse line. **a** Schematic showing knock-in strategy of *Nppa-rtTA* mouse line by homologous recombination. **b** Schematic showing characterization of *Nppa-rtTA* by rtTA responding reporter mice *TetO-LacZ* or *TetO-GFP*. LacZ or GFP is expressed after doxycycline treatment (+Dox). **c** X-gal staining of E12.5 to E14.5 *Nppa-rtTA;TetO-LacZ* heart sections. **d** Whole-mount fluorescence view of *Nppa-rtTA;TetO-GFP* mouse hearts. *Inserts* are bright-field images of same hearts. *Dotted lines* indicate epicardium. **e** Immunostaining for GFP and TNNI3 on *Nppa-rtTA;TetO-GFP* heart sections showed GFP is highly enriched in trabecular myocardium (TM) but sparse in compact myocardium (CM). Each image is a representative of four individual samples. Scale bars, 500 µm (*white* or *black*); 100 µm (*yellow*)

**Fig. 3:**
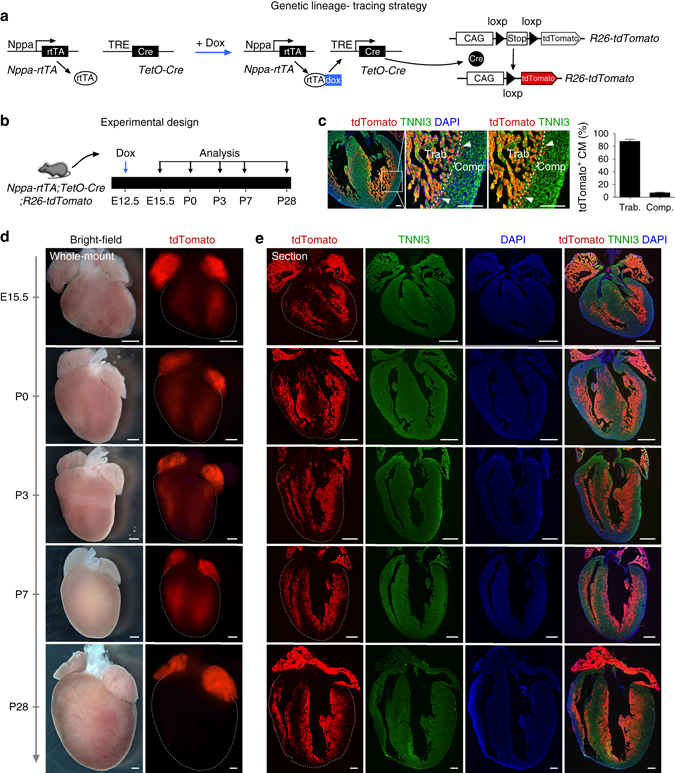
Trabecular myocardium develops into the inner myocardial wall. **a** Schematic diagram showing the genetic labeling strategy of Nppa^+^ cardiomyocytes by tet-on system. After doxycycline (Dox) treatment, rtTA binds to tetO promoter and drives Cre expression. Cre-loxp recombination leads to tdTomato expression. **b** Schematic diagram showing the time point for doxycycline induction and tissue analysis. **c** Immunostaining for tdTomato and TNNI3 on E15.5 heart sections. *Arrowheads* indicate a small number of Nppa^+^ cells (tdTomato^+^) in compact (Comp.) myocardium. *Dotted line* indicates border between trabecular (Trab.) and comp. myocardium. Quantification of the percentage of tdTomato^+^ cardiomyocytes (CM) in Trab. or Comp. layer is shown on the *right panel*. Data are mean ± s.e.m.; *n* = 4. **d** Whole-mount bright-field and fluorescence views of hearts collected from *Nppa-rtTA;tetO-Cre;R26-tdTomato* mice. *Dotted lines* indicate the outline of hearts. **e** Immunostaining for tdTomato and TNNI3 on heart sections. *Dotted lines* indicate epicardium. Nuclei were stained with DAPI. Each image is a representative of four individual samples. Scale bars, 100 µm in **c**; 500 µm in **d**, **e**

To trace the fate of embryonic trabecular cardiomyocytes, we then generated an *Nppa-rtTA* mouse line (Fig. 2a), in which the doxycycline-dependent transactivator rtTA was expressed from the *Nppa* locus. To test if this *Nppa-rtTA* line worked as expected, we crossed the line with rtTA-responsive reporter lines *TetO-nLacZ* ^22^ and *TetO-nGFP* ^23^ (Fig. 2b). By staining for LacZ activity or GFP, we found that rtTA expression was largely restricted to the trabecular myocardium at E12.5 and later time points, but relatively fewer in the compact myocardium of either the left or right ventricles (Fig. 2c–e). We next crossed *Nppa-rtTA* with *TetO-Cre* ^24^ and the reporter *R26-tdTomato* ^25^, permitting inducible lineage tracing of embryonic trabecular myocardium by addition of doxycycline at E12.5 (Fig. 3a, b). To provide a more complete picture of trabecular fate mapping, we analyzed mouse hearts at multiple time points, specifically E15.5, P0, P3, P7, and P28 (Fig. 3b). We first detected E15.5 heart section and found that tdTomato^+^ cardiomyocytes were enriched in the trabecular myocardium, but sparsely in the compact layer (percentage of tdTomato^+^ cardiomyocytes: 87.81 ± 3.29% vs. 6.88 ± 1.10% in trabecular and compact myocardium respectively, Fig. 3c). In doxycycline-treated mice, whole-mount images of *Nppa-rtTA;TetO-Cre;R26-tdTomato* hearts prepared from E15.5 to postnatal day 28 (P28) revealed that tdTomato genetic label was restricted to the subendocardial ventricular myocardium, along with the atrial myocardium (Fig. 3d, e). Within the ventricular myocardium of postnatal hearts, tdTomato^+^ cells were abundant in the sub-endocardial zones of ventricular free walls, extending through roughly one-third to one-half of the mural thickness. Labeling of septal myocardium was less extensive, and was limited to the subendocardial region (Fig. 3d, e). These data demonstrate that embryonic trabecular cardiomyocytes contribute to these labeled sub-endocardial zones of the postnatal ventricular walls. Magnified images showed that the percentage of labeled cardiomyocytes in these portions of the ventricular free walls was high, especially in the papillary muscles (Supplementary Fig. 4), confirming that they mainly originate from pre-existing trabecular myocardium. We noticed that the proportions of labeled cardiomyocytes gradually decreased in the central region of the ventricular walls of postnatal hearts (Supplementary Fig. 4). This mosaic region, which we termed “hybrid myocardium”, likely resulted from Nppa^+^ cells derived from trabecular myocardium mixing with unlabeled cells from compact myocardium. Alternatively, it is possible that this hybrid myocardial region might arise from imprecise marking of the trabecular/compact myocardial boundary by our *Nppa-rtTA;TetO-Cre* tracing strategy. This is less likely, as the labeling of cardiomyocytes at E15.5 is efficient in the trabecular myocardium, and sparse in the compact myocardium (Fig. 3c and Supplementary Fig. 4, E15.5), suggesting the gradual dilution of labeling in the central region of the ventricular walls during perinatal and postnatal development.

### Fetal compact myocardium expands more rapidly than the trabecular meshwork

**Fig. 4:**
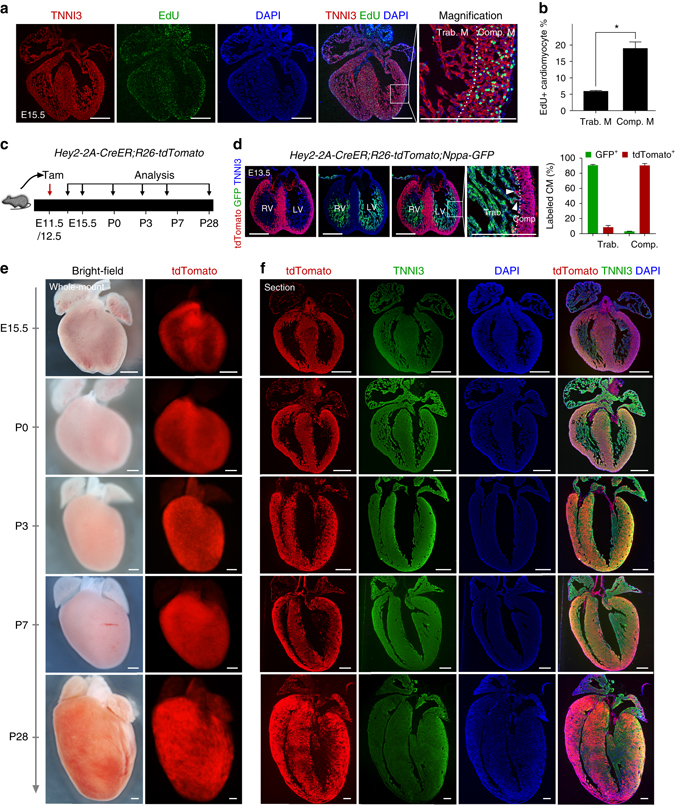
Compact myocardium contributes to the middle and outer myocardial wall. **a** Immunostaining for EdU and TNNI3 on E15.5 heart sections. *Dotted line* in magnified image denotes border between trabecular myocardium (Trab. M) and compact myocardium (Comp. M). **b** Quantification of the percentage of EdU^+^ cardiomyocytes in Trab. M or Comp. M. *P < 0.05; Data are mean ± s.e.m.; *n* = 4. **c** Schematic diagram showing the time point for tamoxifen (Tam) induction and tissue analysis. **d** Immunostaining for tdTomato, GFP, and TNNI3 on *Hey2-2A-CreER;R26-tdTomato;Nppa-GFP* heart section. *Dotted line* indicate border between Trab. and Comp. myocardium. *Arrowheads* indicate tdTomato+ cardiomyocytes (CM) in trab. layer. Quantification of the percentage of labeled CM in Trab. or Comp. is shown on the *right panel*. Data are mean ± s.e.m.; *n* = 4. **e** Whole-mount bright-field and fluorescence views of hearts collected from *Hey2-2A-CreER;R26-tdTomato* mice. **f** Immunostaining for tdTomato and TNNI3 on heart sections. Each image is a representative of four individual samples. Scale bars, 500 µm

To test if unlabeled cells from the compact myocardium expand into and mix with the trabecular myocardium, or alternatively, that labeled cells from trabecular myocardium expand into and mix with the compact myocardium, we first examined the rate of cardiomyocyte proliferation in the compact and trabecular components of the developing heart. By EdU incorporation assay, we detected a robust proliferation of cardiomyocytes in the compact myocardium when compared with that observed in the trabecular myocardium (5.88 ± 0.31 vs. 18.97 ± 1.98% in trabecular and compact myocardium, respectively, Fig. 4a, b). As cardiomyocytes in the compact myocardium proliferate significantly more than those making up the trabeculations, it is more likely that cardiomyocytes of compact myocardium expand into and intermingle with adjacent trabecular myocardium during the late embryonic and postnatal stages.

### Fetal Hey2^+^ cardiomyocytes contribute to the postnatal hybrid myocardial zone

**Fig. 5:**
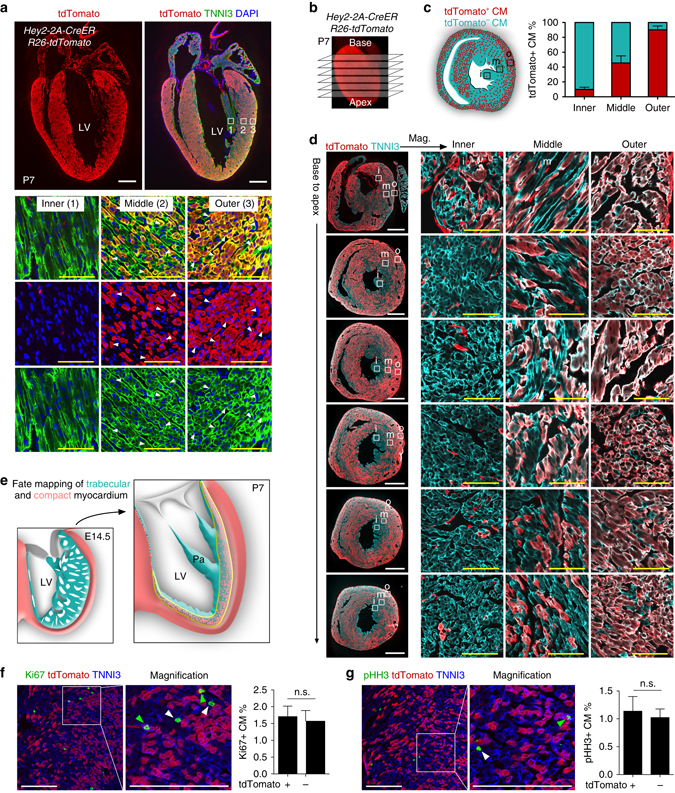
Fetal compact myocardium expands into pre-existing trabecular layer to form hybrid myocardial zone. **a** Immunostaining for tdTomato and TNNI3 on P7 *Hey2-2A-CreER;R26-tdTomato* heart sections. The *boxed regions* in the inner, middle, and outer myocardial wall are magnified. Tamoxifen was induced at E12.5. **b** Transverse sections of P7 heart from base to apex. **c** Quantification of the percentage of tdTomato^+^ cardiomyocytes (CM) in the inner, middle and outer myocardial wall. *n* = 4. **d** Immunostaining for tdTomato and TNNI3 on transverse sections shows different contribution of tdTomato^+^ CMs in the inner i, middle m, and outer o myocardial wall. **e** Cartoon figure showing the fate mapping of embryonic trabecular and compact myocardium into the postnatal stage. *Yellow lines* encircles hybrid myocardium. *LV* left ventricle, *Pa* papillary muscle. **f**, **g** Immunostaining for tdTomato, TNNI3, and Ki67 (**f**) or pHH3 (**g**) on P7 *Hey2-2A-Cre;R26-tdTomato* heart sections. Quantification of the percentage of proliferating cardiomyocytes (CM) in tdTomato^+^ or tdTomato^−^ populations. *n.s*. non-significant; *n* = 4. Scale bars, 500 µm (*white*) in **a**, **d**; 100 µm (*yellow*) in **a**, **d**; 100 µm in **f**, **g**

To assess directly the contributions of fetal compact myocardium to the postnatal ventricular wall, we next crossed the fetal compact myocardial Cre line *Hey2-2A-CreER* (Supplementary Fig. 3a) with *R26-tdTomato* line^25^ to trace the Hey2^+^ cardiomocytes of the embryonic heart, and collected heart samples at different time points for analysis (Fig. 4c). We also generated *Hey2-2A-CreER;R26-tdTomato;Nppa-GFP* mice and collected heart samples at E13.5. Immunostaining for tdTomato and GFP, as a surrogate for Nppa, showed that *Hey2-2A-CreER* mainly labels the compact layer, and minimally the Nppa^+^ trabecular layer, whereas GFP is largely restricted in the trabecular layer and sparsely in the compact layer (percentage of tdTomato^+^ cardiomyocytes: 90.31 ± 2.51 vs. 8.55 ± 2.39% in the compact and trabecular myocardium, respectively; percentage of GFP^+^ cardiomyocytes: 90.45 ± 1.09 vs. 3.05 ± 0.22% in the trabecular and compact myocardium, respectively; Fig. 4d). At E15.5, *Hey2-2A-CreER* had efficiently labeled the compact myocardium and the interventricular septum, but relatively fewer cells in the trabecular meshwork (Fig. 4e, f, E15.5). Analysis of hearts at the later stages of P3, 7, and 28 showed that the compact cardiomyocytes formed the outer layer of the postnatal ventricular walls, with few tdTomato^+^ cells in the papillary muscles or the inner parts of the walls (Fig. 4f). A substantial number of tdTomato^+^ cells were found in the middle part of the wall adjacent to the bases of the papillary muscles (Fig. 5a and Supplementary Fig. 5). Quantification of the number of tdTomato^+^ cardiomyocytes in the different layers of the ventricular walls of P3 heart showed <10% of cardiomyocytes were tdTomato^+^ cells in the inner layer, over half in the middle layer, and over 90% in the outer layer, respectively, taking these layers as representing thirds of the mural thickness (Supplementary Fig. 5c). This suggests that the fetal Hey2^+^ cardiomyocytes form the epicardial layer of the postnatal ventricular walls, and contributes to a substantial part of the hybrid region, but minimally to the endocardial layer, where the papillary muscles are anchored.

As the arrangement of the cardiomyocytes in the ventricular walls is oriented three-dimensionally^26^, we next collected transverse heart sections from P7 *Hey2-2A-CreER;R26-tdTomato* mice, and quantified the number of labeled cardiomyocytes in each section from base to apex (Fig. 5b). Transverse sections at different levels of the P7 heart showed that there were three distinct layers of myocardium in the left ventricle wall: an inner layer with sparse tdTomato^+^ cardiomyocytes, a middle hybrid component with mosaic tdTomato^+^ cardiomyocytes, and an outer layer with mostly tdTomato^+^ cardiomyocytes (tdTomato^+^ cardiomyocytes: 9.95 ± 0.58, 45.45 ± 0.58, and 90.15 ± 5.20% in the inner, middle, and outer layer, respectively, Fig. 5c, d), consistent with the data revealed by the coronal sections (Supplementary Fig. 5). The *Hey2-2A-CreER* had labeled about half of the cardiomyocytes in the middle hybrid region of the left ventricular free walls. These data indicate the presence of a hybrid myocardial zone formed by Nppa^+^ and Hey2^+^ cardiomyocytes that mainly originate from the embryonic trabecular and compact myocardial regions (Fig. 5e). Immunostaining for Ki67 and pHH3 showed that there is no significant difference in the percentage of proliferating cardiomyocytes between tdTomato^+^ and tdTomato^–^ cardiomyocytes (Fig. 5f, g), indicating these two populations in the hybrid zone expand equally at P7. Detailed gene profile of these tdTomato^+^ and tdTomato^–^ cardiomyocytes in the hybrid zone remains a topic for further investigation.

**Fig. 6:**
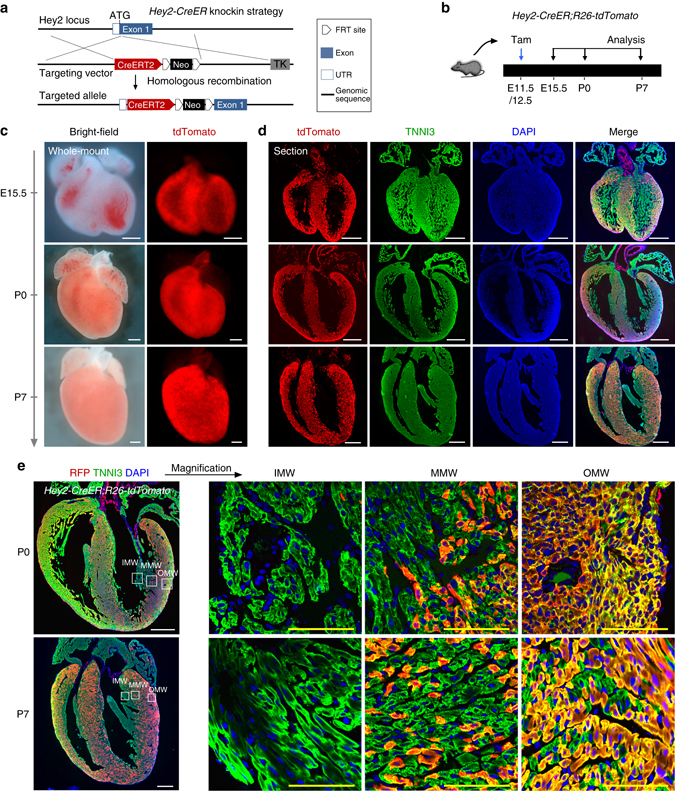
Fate mapping of fetal compact myocardium. **a** Schematic diagram showing strategy for generation of *Hey2-CreER* knock-in allele. **b** Schematic diagram showing the time point for tamoxifen induction (Tam) and tissue analysis. **c** Whole-mount bright-field and fluorescence views of hearts collected from *Hey2-CreER;R26-tdTomato* mice. **d** Immunostaining for tdTomato and TNNI3 on heart sections. Nuclei were stained with DAPI. **e** Magnified images showing inner myocardial wall (IMW), middle myocardial wall (MMW) and outer myocardial wall (OMW). Each image is a representative of four individual samples. Scale bars, 500 µm (*white*); 100 µm (*yellow*)

To confirm the above lineage tracing data based on *Hey2-2A-CreER* line, which was generated by CRISPR/Cas9 technology^27^, we also generated a conventional knock-in *Hey2-CreER* line by recombination in ES cells, in which cDNA encoding CreER fusion protein was inserted into ATG of endogenous Hey2 gene (Fig. 6a). CreER detected by ESR staining showed its expression pattern in the compact myocardium from E10.5 through E12.5 (Supplementary Fig. 6), consistent with the *Hey2* in situ hybridization data (Fig. 1b). We treated mice with tamoxifen and collected hearts at later stages (Fig. 6b). Lineage tracing experiments based on the *Hey2-CreER;R26-tdTomato* line revealed expansion of the pre-labeled compact myocardium during development of the ventricular walls (Fig. 6c, d). In neonatal hearts, Hey2-derived cells make up the majority of cardiomyocytes in the outer part of the ventricular walls, about half in the middle parts, and few in the inner components (Fig. 6e).

### The middle part of postnatal ventricular wall includes fetal trabeculations

The evolution of the trabecular meshwork occurs concomitant with the formation of coronary vessels during the perinatal period^28^. In the embryonic heart, endocardial cells contribute to a substantial number of coronary endothelial cells^29–31^. During perinatal development, these endocardial cells become entrapped in the trabecular meshwork during a process in which the intertrabecular spaces are closed, and the myocardium is consolidated^32^. To explore this accompanying cellular event further, we used an endocardium inducible Cre line, *Npr3-CreER* ^33^. In situ hybridization data demonstrated that *Npr3* was specifically expressed in the endocardium from E12.5 to E14.5 (Supplementary Fig. 7a). Tamoxifen induction of *Npr3-CreER;R26-tdTomato* mice in this window had labeled the atrium and the inner parts of the ventricular walls by P7 (Supplementary Fig. 7b). These endocardium derived cells contributed to the majority of the coronary vessels in the inner, but not the outer, ventricular walls of the postnatal heart (Supplementary Fig. 7c), suggesting entrapment of endocardial cells between the coalescing trabeculations during the perinatal period. We also noticed that endocardial cells contribute to the coronary vessels in the middle part of ventricular wall (Supplementary Fig. 7c), indicating coalescence of trabecular myocardium in this region. To validate this, we utilized a *Fabp4-Cre* line that labels coronary vascular endothelial cells, but not the endocardium^34, 35^. Using P7 *Fabp4-Cre;R26-tdTomato* mice, we could distinguish between endocardial cells and coronary endothelial cells, both by expression of the coronary endothelial cell lineage marker (tdTomato), and by the characteristically different morphology of endocardium as opposed to coronary microvasculature (Supplementary Fig. 7d, e). Although most endocardial cells had already adopted a coronary endothelial cell fate at P7, a subset of endocardial cells remained in the intertrabecular spaces, which extended deep into the middle region of ventricular wall (Supplementary Fig. 7d–f). The presence of this endocardial remnant in the hybrid region of the postnatal ventricular wall suggests that this region in postnatal heart was once trabeculated and wrapped in endocardium at embryonic stages. The middle part of the postnatal ventricular wall, therefore, includes portions of the trabecular myocardium of the embryonic heart.

### Sema3a^+^ cells form the innermost layer of the postnatal ventricular wall

**Fig. 7:**
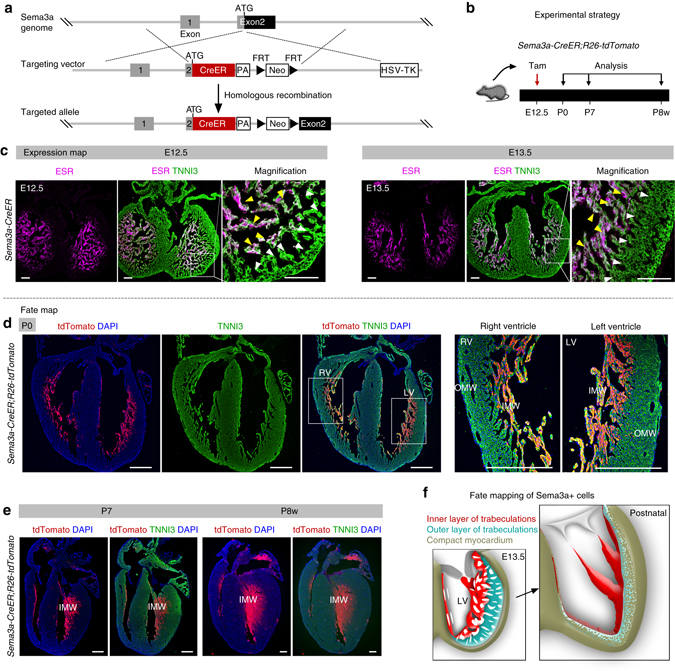
Sema3a^+^ cells contribute to the innermost layer of ventricle wall in postnatal heart. **a** Schematic showing strategy for generation of *Sema3a-CreER* knock-in allele by homologous recombination. **b** Experimental strategy for tamoxifen administration and tissue analysis. **c** Immunostaining for ESR and TNNI3 on E12.5 and E13.5 *Sema3a-CreER* hearts. Most Sema3a^+^ (ESR^+^) cells were detected in inner trabecular layer (*yellow arrowheads*) but not in outer trabecular layer (*white arrowheads*). **d**, **e** Immunostaining for tdTomato and TNNI3 on heart section from P0, P7, and P8w (8 weeks) *Sema3a-CreER;R26-tdTomato* hearts. Sema3a-derived cells are restricted in the innermost layer of ventricle wall (IMW) in postnatal heart. **f** Cartoon image showing cell fate of Sema3a^+^ cells in postnatal heart. Scale bars, 100 µm in **c**; 500 µm in **d**, **e**; Each image is representative of four individual samples

To examine further the potential tripartite configuration of the postnatal ventricular walls, we generated a new genetic tool, namely a *Sema3a-CreER* knock-in allele, to follow the fate of the innermost trabecular cardiomyocytes of the postnatal heart (Fig. 7a, b). At E12.5 and E13.5, Sema3a/CreER was largely expressed in the luminal portions of the meshwork trabecular myocardium, but rarely in the portions where they joined the compact myocardium (Fig. 7c). Pulse labeling of Sema3a-expressing cells at E12.5, followed by chase into the postnatal heart, showed that the majority of Sema3a lineage of inner trabecular myocardium contributed primarily to the innermost sub-endocardial layer and the papillary muscles, but made minimal contributions to the middle or outer (sub-epicardial) layers of the ventricular walls (Fig. 7d, e). Fate mapping data from Nppa, Hey2, Sema3a, as well as endocardial and coronary vascular lineage tracings, therefore shows that the innermost Sema3a^+^ trabeculations make up the sub-endocardial layer of the mature ventricle wall, including the papillary muscles, whereas the hybrid myocardium is derived from the compact myocardium, along with the adjacent portions of trabecular myocardium (Fig. 7f).

### Clonal analysis of Hey2^+^ cardiomyocyte by single cell lineage tracing

**Fig. 8:**
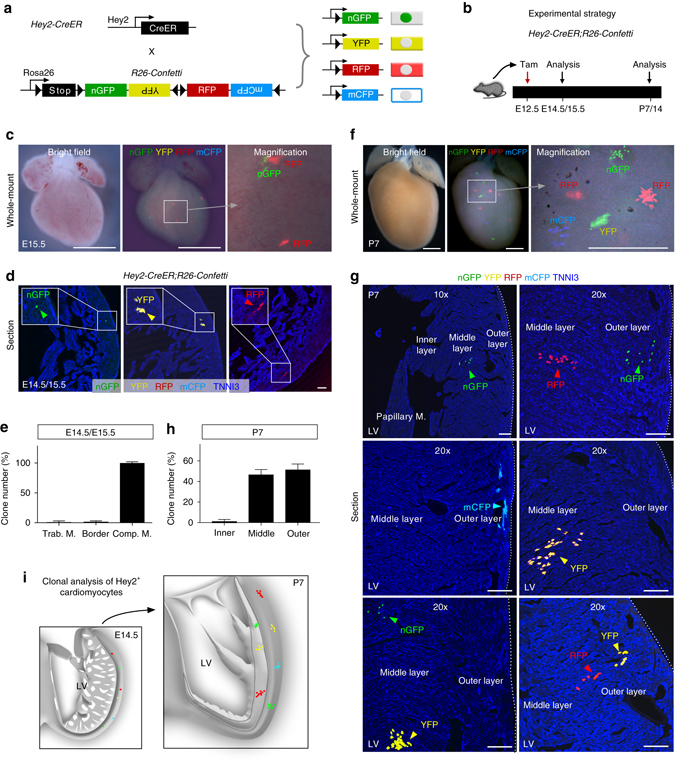
Clonal analysis of single Hey2^+^ cardiomyocyte from fetal to postnatal stage. **a** Schematic figure showing four alternative recombination results (nGFP, YFP, RFP, and mCFP) by crossing *Hey2-CreER* with *R26-Confetti* line. **b** Schematic figure showing experimental strategy. **c**, **f** Whole-mount fluorescence view of E15.5 and P7 *Hey2-CreER;R26-Confetti* hearts. **d**, **g** Sectional view of clones in E14.5/E15.5 and P7 *Hey2-CreER;R26-Confetti* heart sections. *Arrowheads* indicate single color colonies. *Dotted lines* in **g** indicate epicardium. **e**, **h** Quantification of the percentage of clone numbers in the inner, middle, and outer layer in each heart. *n* = 18 for E14.5/E15.5; *n* = 51 for P7. **i** Cartoon image showing sparse labeling of Hey2^+^ cells at embryonic stage and their expansion and contribution to the middle and outer myocardium layer of postnatal heart. Scale bars, 1 mm in **c**, **f**; 100 µm in **d**, **g**

During Hey2-CreER lineage tracing, we noticed that 8.55 ± 2.39% of cardiomyocytes in the trabecular myocardium are tdTomato^+^ (close to the border between trabecular and compact myocardium). As genetic lineage tracing depends on the gene promoter that drives Cre recombinase, it is technically challenging to achieve clear cut border between two different myocardial layers. The labeling of Hey2^+^ cardiomyocytes in the trabecular layer of fetal heart may confound the interpretation of fate mapping result at postnatal stage. To further validate if the compact myocardial cells of fetal heart contribute to the hybrid middle zone in postnatal ventricular wall, we employed the single cell clonal analysis through sparse labeling by the R26-Confetti strategy^36^. Crossing of *Hey2-CreER* with *R26-Confetti* allele resulted in four exclusive recombination readouts: nuclear GFP (nGFP), YFP, RFP, and membrane CFP (mCFP) in Hey2-expressing cells (Fig. 8a). We injected tamoxifen at low dosage to achieve sparse labeling at E12.5, and collected heart samples at later stages for analysis (Fig. 8b). Whole-mount and sectional fluorescence views of *Hey2-CreER;R26-Confetti* heart samples showed very sparse labeling of cardiomyocytes in the compact myocardial wall (Fig. 8c, d). Almost all of these labeled cardiomyocytes are restricted to the compact layer, with very sparsely labeled cells in the trabecular layer or the border region between the compact and trabecular layers (Fig. 8d, e). In P7 *Hey2-CreER;R26-Confetti* heart, we found several isolated clones with multiple cells in each clone, and all cells in individual clone were of a single color (Fig. 8f). About half of the clones were within the middle layer, whereas the other half of the clones were located in the outer layer, with very few clones detected in the inner layer of the ventricular wall (Fig. 8g, h). These data from clonal analysis, therefore, demonstrated that Hey2^+^ cardiomyocytes in the embryonic compact myocardium expand and contribute to the hybrid middle layer of the postnatal ventricular walls (Fig. 8i).

### Inhibition of Hey2^+^ cell expansion produces a non-compacted hybrid zone

**Fig. 9:**
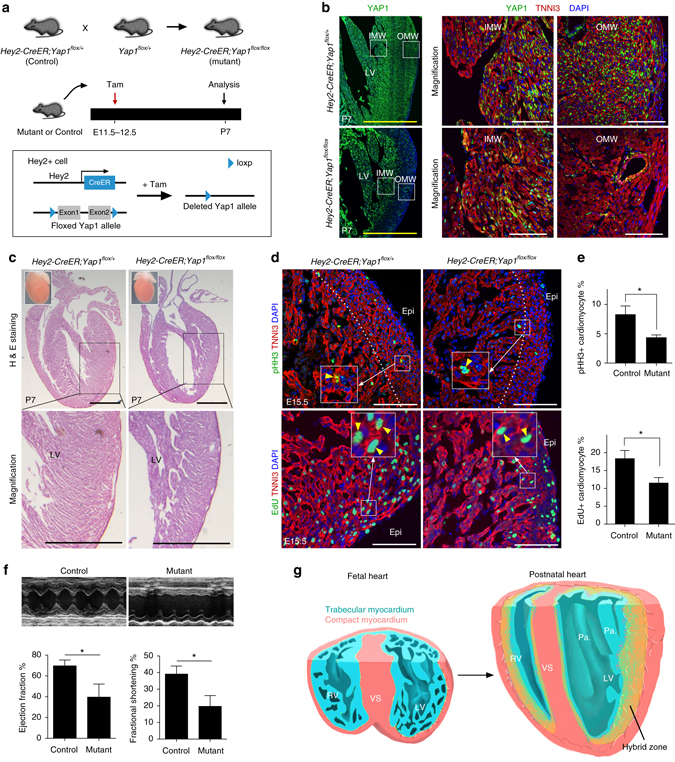
Inhibition of fetal compact myocardial expansion results in prominent trabeculae and thin compacted layer of postnatal heart. **a** Schematic figure showing generation of Yap1 gene deletion in Hey2^+^ cells by tamoxifen injection at embryonic stage. **b** Immunostaining for YAP1 and TNNI3 on postnatal day 7 (P7) control and mutant heart sections. Although YAP1 is detected in the inner myocardial wall (IMW) of both control and mutant hearts, YAP1 is reduced in the outer myocardial wall (OMW) of mutant heart compared with that in the control heart. **c** Hematoxylin and eosin (H&E) staining of heart sections from P7 control (*left*) and mutant (*right*) mice. *Inserts* indicate whole-mount images of hearts. **d** Immunostaining for TNNI3 and pHH3 or EdU on control and mutant heart sections. *Yellow arrowheads* indicate proliferating cardiomyocytes in the magnified inserts. **e** Quantification on the percentage of pHH3^+^ or EdU^+^ cardiomyocytes is shown on the *right panel*. **f** Echocardiographic analysis of heart function showed a significant reduction of ejection fraction and fractional shortening in the mutant, compared with the control. *P < 0.05; *n* = 4 for mutant and *n* = 5 for control. **g** Cartoon image showing hybrid myocardial zone in the postnatal heart derived from trabecular layer and compact myocardium. The hybrid zone (*yellow*) is interposed between the inner myocardial wall (*blue*) produced mainly by trabecular coalescence and the outer myocardial wall (*red*) produced by compact myocardial expansion. *RV* right ventricle, *LV* left ventricle, *VS* ventricular septum; *Pa*. papillary muscle. Scale bars, 1 mm (*black* or *yellow*); 100 µm (*white*)

As fetal compact myocardium expands and intermingles with trabecular myocardium to form the middle hybrid myocardium of the postnatal heart, we hypothesized that inhibiting expansion of fetal compact myocardium would result in abnormal formation of the hybrid myocardium. To inhibit the expansion of fetal compact myocardium, we ablated Yap1, a key transcription co-factor, which is required for normal cardiomyocyte proliferation during cardiac development^37–39^, in fetal compact cardiomyocytes using *Hey2-CreER* and a conditional Yap1 allele, *Yap1* ^*flox*^. *Hey2-CreER;Yap1* ^*flox/flox*^ embryos were treated with tamoxifen at E11.5 and E12.5, and heart samples were collected at P7 (Fig. 9a). Immunostaining for YAP1 on E15.5 samples showed that, whereas YAP1 expression was readily detected in trabecular myocardium, it was not detected in the majority of cardiomyocytes in the compact myocardium (Supplementary Fig. 8b). Immunostaining for YAP1 on P7 heart sections showed that, whereas YAP1 expression was readily detected in the inner myocardial wall (IMW), it was significantly reduced in the outer myocardial wall (OMW) of *Hey2-CreER;Yap1* ^*flox/flox*^-mutant mice compared with *Hey2-CreER;Yap1* ^*flox/+*^ littermate controls (Fig. 9b), indicating specific ablation of Yap1 in the fetal compact myocardium. As assessed using hematoxylin and eosin (H&E) staining, there was extensive non-compacted myocardium in the hybrid zone of mutant hearts, but not in control hearts (Fig. 9c). The formation of the innermost layer, including the papillary muscles, in the mutant hearts, however, was indistinguishable from that of controls (Fig. 9c). Immunostaining for proliferation marker pHH3 and EdU incorporation showed significantly reduced proliferation of compact myocardial cardiomyocytes in the mutant hearts compared with that of controls at E15.5 (pHH3^+^ cardiomyocytes: 8.28 ± 1.46 vs. 4.35 ± 0.45%; EdU^+^ cardiomyocytes: 18.35 ± 2.29 vs. 11.50 ± 1.54% in the mutants and controls, respectively, Fig. 9d, e and Supplementary Fig. 8). The detection of proliferating cardiomyocytes in the mutant heart might be due to the compensatory growth of those Yap1^+^ cardiomyocytes in the compact myocardium, as inducible Yap1 deletion by *Hey2-CreER* is not complete in all Hey2^+^ cardiomyocytes in our study. Examination of trabecular marker connexin 40 (Cx40) or compact marker Hey2 on the mutant heart section, however, showed that the trabecular or compact myocardial identity remains similar in the mutants compared with the controls (Supplementary Fig. 9). Echocardiography of adult mutant and control hearts showed a significant reduction of ejection fraction (EF) and fractional shortening (FS) in the mutant hearts compared with the controls (EF: 69.66 ± 5.56 vs. 39.60 ± 12.55%; FS: 39.12 ± 4.86 vs. 19.71 ± 6.45%; respectively, Fig. 9f), indicating reduction of heart function in the mutants. Taken together, these data indicate that expansion of Hey2^+^ cardiomyocytes is essential for formation of the hybrid myocardial zone (Fig. 9g).

### Deletion of Yap1 in trabecular layer does not produce a non-compacted hybrid zone

**Fig. 10:**
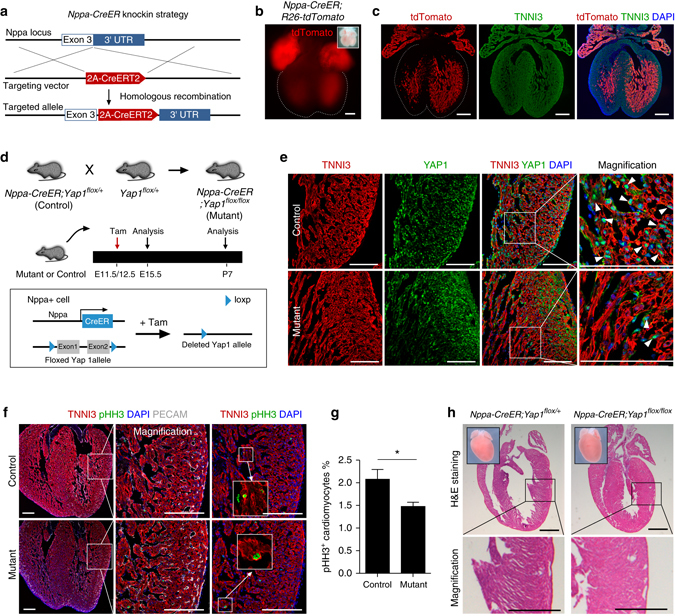
Deletion of Yap1 in fetal trabecular myocardium does not result in thin compact myocardium. **a** Schematic figure showing generation of *Nppa-CreER* knock-in allele. **b** Whole-mount fluorescence view of E15.5 *Nppa-CreER;R26-tdTomato* heart. Tamoxifen was injected at E12.5. **c** Immunostaining for tdTomato and TNNI3 on heart section showed *Nppa-CreER* mainly targets trabecular myocardium. **d** Schematic figure showing strategy for Yap1 knockout and experimental design. **e** Immunostaining for YAP1 and TNNI3 on control or mutant heart sections shows a significant reduction of YAP1^+^ cardiomyocytes (*arrowheads*) in the mutant compared with the control. **f** Immunostaining for pHH3, TNNI3 and PECAM on the E15.5 mutant or control heart sections. **g** Quantification of the percentage of pHH3^+^ cardiomyocytes in trabecular myocardium of the control and mutant heart samples. *P < 0.05; *n* = 4. **h** Hematoxylin and eosin (H&E) staining of heart sections from P7 control and mutant hearts. *Inserts* indicate whole-mount images of hearts. Scale bars, 1 mm in **h**; 200 µm in **b**; 100 µm in **c**, **e**, **f**

To further explore if deletion of Yap1 in trabecular layer would yield the non-compaction phenotype, we generated *Nppa-CreER* knock-in allele in which a CreER is controlled by *Nppa* regulatory elements (Fig. 10a). Tamoxifen induction at E11.5-E12.5 in *Nppa-CreER;R26-tdTomato* specifically labeled the cardiomyocytes in atrial walls and the trabecular layer of the ventricular walls at E15.5 (Fig. 10b, c). To delete Yap1 in fetal trabecular myocardium, we crossed *Nppa-CreER* with *Yap1* ^*flox*^ allele to generate *Nppa-CreER;Yap1* ^*flox/flox*^ mice (Fig. 10d). Tamoxifen induction at E11.5-E12.5 ablated Yap1 in the majority of the trabecular cardiomyocytes (Fig. 10e). Immunostaining for the proliferation marker pHH3 showed that there was a significant reduction of proliferating cardiomyocytes in the trabecular myocardium of E15.5-mutant hearts, when compared with their littermate controls (1.49 ± 0.10 vs. 2.09 ± 0.21%; respectively, Fig. 10f, g). Analysis of P7 hearts by H&E staining, however, showed that there was no observable non-compacted hybrid zone or thin ventricular wall phenotype in the mutants, as compared with the littermate controls (Fig. 10h). These data show that deletion of Yap1 in the trabecular myocardium does not generate non-compacted myocardium. It is possible that expansion of compact myocardium to compensate for the reduced proliferation of cardiomyocytes in the trabecular myocardium at later embryonic or neonatal stages, or the compensatory growth of trabecular cardiomyocytes (e.g., from a few remaining Yap1^+^ cardiomyocytes), or Yap1 is not required in the less-proliferating cardiomyocytes in trabecular myocardium for compaction process.

## Discussion

There has been a long debate as to how trabeculations forming the inner ventricular walls of the developing heart aggregates together to produce the compact myocardium of the mature ventricular free walls. Two major models have been advanced to explain this potential consolidation of trabecular myocardium into compact muscle (Supplementary Fig. 1a): expansion of compact myocardium into the trabecular layer (model 1), or coalescence of trabecular muscles by themselves (model 2). In both of these two models, compact myocardium expands by itself to form an increasingly thicker ventricular wall during cardiac development. The difference between these two models is the fashion in which the trabecular portion is transformed into compacted wall. To resolve this question, we have now used genetic lineage tracing to track the fates of the Nppa^+^ and Hey2^+^ cardiomyocytes, mainly representing fetal trabecular and compact myocardial components, into the postnatal heart. The fate mapping results, based on Nppa, Hey2, and Sema3a-driven genetic labeling, demonstrate that the luminal portions of the initial trabeculations form the sub-endocardial portion of the postnatal ventricular wall. The portions of the Nppa^+^ cardiomyocytes adjacent to the compact myocardium, along with derivatives of the embryonic compact myocardium itself, then intermingle to form a middle region of the mature ventricular wall, which we have dubbed the hybrid myocardial zone. The embryonic compact myocardium itself then expands significantly during late embryonic and postnatal stages so as to form the thickened subepicardial layer of the mature ventricular wall (Supplementary Fig. 10). The expansion of Hey2^+^ compact cardiomyocytes of fetal heart into the trabecular layer to form the hybrid zone of the postnatal heart was also supported by the single cell clonal analysis. These fate mapping studies, therefore, show that our new model is a combination of the two previously proposed models, differing on the basis of our novel identification of this middle hybrid zone (Supplementary Fig. 10). When forming the middle hybrid myocardial zone, it is more likely that the compact myocardium expands inward, rather than the initial trabeculations expanding outward, as cardiomyocytes in fetal compact myocardium proliferate at a significantly greater rate than those making up the trabecular layer. Our lineage tracing data based on identification of the ventricular endocardium, as apposed to the coronary endothelial cells, provided additional evidence that the anatomic location of hybrid myocardium of postnatal heart could be traced back to the luminal components of the trabecular layers of the fetal heart. Although there is relatively less proliferation rate in trabeculations, their volume expands with the contribution of proliferating cardiomyocytes from compact layer, thus facilitating trabecular coalescence for compaction (Supplementary Fig. 10). During this trabecular coalescence, trapped endocardial cells give rise to coronary endothelial cells^28^.

Our study also provides new evidence that the expansion of compact myocardium is essential for subsequent trabecular growth, and its coalescence into compact myocardium. Inhibition of compact myocardial proliferation by YAP1 ablation resulted in aberrant formation of the hybrid myocardial zone, with abnormal persistence of trabeculations and multiple intertrabecular spaces. These functional data indicate that the trabecular compaction not only involves the trabecular cardiomyocytes through a process of coalescence, but also requires the contribution of fetal compact myocardium. Inhibition of expansion of the fetal compact myocardium also resulted in thinning of the compact ventricular myocardium, whereas the papillary muscles were formed in normal fashion. This is consistent with the phenotypic features of symptomatic individuals with non-compaction cardiomyopathy, in which excessive trabeculations are associated on occasion with thinning of the compact myocardium, but without severe defects in the papillary muscles^7, 8^. Our study thus suggests that noncompaction may not be attributed to a defect exclusively involving the trabeculations themselves. The compact myocardium continues to contribute substantially to formation of a major part of the ventricular wall in the late embryonic and early neonatal stages, with compression of intertrabecular spaces serving to form the middle hybrid zone of the ventricular wall.

Most studies have suggested that noncompaction is persistence of excessive trabeculation rather than reduced resolution of compaction. Our study now suggests that, in addition to potential excessive trabeculation, there is a specific morphogenic process that may be perturbed, which then recapitulates the phenotypic features. The contribution made by the Hey2^+^ lineages of compact myocardium to the trabecular layer expands the myocardial volume, compressing the intertrabecular spaces in this middle region and consolidating the walls concomitant with the formation of the coronary vasculature. Re-examination of the anatomic features of many individuals with the noncompaction phenotype permits recognition of a tripartite configuration of the ventricular walls^7, 8, 15^, in which the middle part of the wall retain a lace-like configuration, with deep intertrabecular spaces as part of the meshwork. In addition, thinning of the compact myocardium is typically a feature of symptomatic individuals having excessive trabeculations^9, 16, 17^, and also in most animal models thus far considered to show the non-compaction cardiomyopathy^18, 19^. These lines of evidence emphasize the critical role of proliferation and expansion of Hey2^+^ lineages of compact layer for trabecular compaction. The identification of this middle hybrid component of the myocardial wall, as well as the contribution of compact myocardium to the phenotype of excessive trabeculations, provides a basis for further understanding the pathophysiology of noncompaction. The genetic tools generated in this study have now helped to dissect the molecular mechanisms regulating trabecular compaction. Recent work showed that Dll4-Notch1 signaling induce trabecular formation and later coordinate its patterning and compaction with coronary vessel formation to generate the mature chamber^40, 41^, so it remains a fascinating question of how Hey2^+^ or Nppa^+^ lineages are directly affected in Dll4-Notch mutant. More broadly, it will be important to understand in detail how different signaling pathways converge to regulate the trabecular compaction, such as the Dll4-Notch^40, 41^, Mib1^17^, BMP^42^, and TGF-β1^19^ pathways, all of which have previously been proposed to be involved in the LVNC.

## Methods

### Mice

All mice were used in accordance with the guidelines of the Institutional Animal Care and Use Committee of the Institute for Nutritional Sciences, Shanghai Institutes for Biological Sciences, Chinese Academy of Sciences. Mice were maintained on a C129/C57BL6/J mixed background. Both male and female mice were randomized in different experiment groups in this study. Doxycycline (Sigma) was dissolved in 50% ethanol and introduced by gavage at indicated time (0.2–0.25 mg/g). Tamoxifen was dissolved in corn oil, 4-hydroxytamoxifen (Sigma) was dissolved in 25% ethanol-corn oil, and introduced by gavage at indicated time (0.01–0.1 mg/g)^43^. *TetO-LacZ*, *TetO-GFP*, *TetO-Cre, Fabp4-Cre*, *R26-tdTomato, R26-Confetti*, and *YAP1* were described previously^22–25, 34, 36, 44^. *Nppa-GFP*, *Nppa-rtTA*, *Hey2-CreER*, and *Sema3a-CreER* lines were generated by homologous recombination using Red/ET recombineering as previously described^45^. For *Nppa-GFP* and *Nppa-rtTA* mouse lines, cDNAs-encoding DTRGFP fusion protein^46^, or rtTA were inserted into frame with the translational start codon of the Nppa gene. For Nppa-CreER allele, a cDNA encoding Cre recombinase fused with a mutant form of the estrogen receptor hormone-binding domain (CreER^T2^)^47^ was inserted into the translational stop codon of Nppa gene (before 3′ UTR), with a self-cleaving 2 A peptide sequence linking Nppa and CreER. For *Hey2-CreER*, *Npr3-CreER*, and *Sema3a-CreER* mouse lines, a cDNA encoding Cre recombinase fused with a mutant form of the estrogen receptor hormone-binding domain (CreER^T2^)^47^ was inserted in frame with the translational start codon of the Hey2, Npr3, or Sema3a gene. After G418 selection, over 100 clones were selected for retrieval of genomic DNA and screening of positive clones. To screen the correct targeted clones, we used long PCR assays with primer pairs spanning the targeting vector and flanking genomic DNA according to standard protocols^48^. After validation of correct targeting and normal karyotype, we expanded at least three positive ES clones for each line and injected ES cells into blastocysts for mice generation. The obtained chimeric mouse lines were crossed to C57B/6 lines for germline transmission. *Hey2-2A-CreER* line was generated by homologous recombination using CRISPR/Cas9 technology (gRNA sequence: 5′-AGATTCAAGAATAAGTTAAA-3′ and 5′-GTAACTGATGTCGTCCATTT-3′) according to published protocols^49^. For *Hey2-2A-CreER* line, a cDNA encoding CreER^T2^ followed by a polyadenylation sequence was inserted into the last coding exon of Hey2 gene. A P2A peptide sequence was included in the targeting vector to link Hey2 coding region (before translational stop codon) and CreER^T2^ cDNA, thus allowing expression of both Hey2 and CreER^T2^ proteins. These seven knock-in mice lines (*Nppa-GFP*, *Nppa-rtTa*, *Nppa-CreER*, *Hey2-CreER*, *Hey2-2A-CreER*, *Npr3-CreER*, and *Sema3a-CreER*) were generated by Shanghai Biomodel Organism Science & Technology Development Co. Ltd.

### Electron microscopy

The preparation of samples for electron microscopy was done according to protocols described previously^50^. Briefly, embryonic hearts from timed pregnancies and neonatal hearts of P0, P3, and P7 were dissected in PBS and fixed overnight in 4% paraformaldehyde (PFA) in PBS at 4 °C. After wash with PBS for three times, hearts were transferred to 30% sucrose for overnight cryopreservation at 4 °C and embedded in OCT. Blocks were sectioned to reveal the transverse plane of ventricle. The blocks were dissolved in PBS at room temperature and post-fixed in 5% acetic acid, 3.7% formaldehyde, and 50% ethanol. Then the sectioned hearts were dehydrated in gradient concentrations of ethanol from 50 to 100% at successive 10% increment. Liquid carbon dioxide was used for critical point drying. The dry specimens were mounted on stubs and sputter-coated with gold before observation with a FEI Quanta 250 scanning electron microscope.

### RNA in situ hybridization

Whole-mount RNA in situ hybridization was carried out as described previously^51^. Briefly, whole-dissected embryonic hearts were fixed in 4% PFA in DEPC-treated PBS overnight. After washing in diethyl pyrocarbonate (DEPC)-treated PBS, fixed embryonic hearts were dehydrated through a graded methanol (25, 50, 75, and 100% methanol in DEPC-treated PBS) for 5 min each on ice with rotation. Treated samples could be stored at −20 °C for a couple of weeks. After dehydration, the embryonic hearts should be rehydrated through 100, 75, 50, and 25% methanol, and rinsed in DEPC-treated PBS. After bleached in 6% hydrogen peroxide that was diluted in DEPC-treated PBS, the embryonic hearts were digested with Proteinase K at a concentration of 20 µg/ml for 10 to 30 min at room temperature. Embryonic hearts were re-fixed with 0.2% glutaraldehyde and 4% PFA for 20 min at room temperature, followed by washing three times with 0.1% tween20 in PBS for 5 min each time and incubation in hybridization buffer for 1 h at 70 °C. Then embryonic hearts were transferred into fresh hybridization buffer containing 1 µg/ml digoxigenin UTP-labeled RNA probes and incubated overnight with rocking at 70 °C. After washing in different wash solution buffers and being treated with ribonucleases to remove redundant probes, the embryonic hearts were incubated with anti-digoxigenin alkaline phosphatase (AP)-conjungated antibody (Roche, 11093274910, 1:2000) overnight with rocking at 4 °C. The embryonic hearts were washed with 2 mM levamisole in mixture of Tris-buffered saline and Tween 20 (TBST) for a whole day to remove the residual antibody and inhibit endogenous AP activity. AP activity for probe detection was developed in BM purple (Roche, 11442074001) in dark until the color has been developed to the desired extent. Sectional in situ hybridization was carried out according to published protocols^52^. In detail, dissected embryos were fixed overnight in 4% paraformaldehyde and embedded in optimal cutting temperature (OCT, Sakura). Embryos were sectioned at 10 µm thickness. Slides were hybridized overnight with 1 µg/ml probes at 65 °C. After washing in salt sodium citrate (SSC) buffer and treated with ribonucleases at 37 °C for 30 min, slides were incubated with alkaline phosphatase coupled anti-digoxigenin antibodies overnight. BCIP/NBT (Promega, S3771) were used to develop the color in dark to the desired extent. Then slides were mounted with glycerol and images were acquired by Olympus microscope (BX53). The primers used for in situ hybridization were listed in Supplementary Table 1.

### X-gal staining

Embryonic hearts from timed pregnancies were fixed in LacZ fix solution (0.2% glutaraldehyde, 5 mM EGTA, 100 mM MgCl_2_ in PBS) for 10 to 20 min based on the size. After washing three times for 15 min in LacZ wash buffer containing 2 mM MgCl_2_, 0.01% sodium deoxycholate, 0.02% NP-40 in 100 mM sodium phosphate buffer, specimens were embedded in OCT. Embryonic hearts were sectioned at 10 µm thickness. Slides were stained in LacZ stain solution containing 1 mg/ml 5-bromo-4-chloro-3-indolyl β-d galactopyranoside (X-gal) in LacZ wash buffer at 37 °C to the desired extent. Then slides were mounted with glycerol and images were acquired by Olympus microscope (BX53).

### Immunostaining

Immunostaining was performed as according to standard protocols^53^. In detail, embryos and postnatal hearts were collected and washed in PBS on ice, and then fixed in 4% PFA at 4 °C for 15 min to 1 h based on the size of tissues. After three washings in PBS, embryos and hearts with fluorescence reporters were observed and photographed using Zeiss stereomicroscope (Zeiss AXIO Zoom. V16). The embryos and hearts were dehydrated in 30% sucrose in PBS overnight at 4 °C, then embedded in OCT and snap frozen. Cyrosections collected at 10 μm thickness were air dried for 30 min to 1 h at room temperature. Tissues were blocked with PBS supplemented with 0.2% triton X-100 and 5% normal donkey serum (Jackson ImmunoResearch, 017-000-121) for 30 min at room temperature, followed by first antibody incubation at 4 °C overnight. We used commercial antibodies for detection of endogenous antigens: GFP (Invitrogen, A21311, 1:200), ESR (Abcam, ab27595, pre-diluted), tdTomato (Rockland, 600-401-379, 1:1000), TNNI3 (Abcam, ab56357, 1:200), YAP1 (Cell signaling technology, 4912 S, 1:200), pHH3 (Millipore, 06-570, 1:1000), Ki67 (Thermo scientific, RM-9106-S0, 1:100), PECAM (BD PHharmingen, 553370, 1:500), Cx40 (Alpha Diagnostic International, CX40-A, 1:100), and EdU (Invitrogen, C10337). Signals were developed with Fluorescence-conjugated Alexa donkey anti rabbit and goat secondary antibodies (Invitrogen, 1:1000) for 30 min at room temperature. For weak signals, we used HRP-conjugated or biotin-conjugated antibodies with tyramide signal amplification kit (PerkinElmer)^54^. Before coverslip addition, tissues were counterstained with mounting medium with 4′6-diamidino-2-phenylindole (DAPI, Vector lab, H-1200). Immunostaining images were acquired by Olympus fluorescence microscope (BX53), Zeiss stereomicroscope (AXIO Zoom. V16), Zeiss confocal laser scanning microscope (LSM510) and Olympus confocal microscope (FV1200). Quantification of proliferating cardiomyocytes was done by an analyzer who was blinded to the experimental groups.

### Whole-mount estrogen receptor staining

Whole-mount estrogen receptor (ESR) staining was performed according to the protocol for whole-mount PECAM staining^55^. Briefly, embryos were collected in PBS on ice and fixed in 4% PFA in PBS overnight at 4 °C. After washing three times in PBS, embryos were dehydrated through a methanol gradient (25, 50, 75, and 100% methanol in PBS) for 15 min each at room temperature. Embryos were bleached in 5% hydrogen peroxide in pure methanol for 2 h at 4 °C to inhibit endogenous peroxidase activity. Then the embryos were rehydrated through 100, 75, 50, and 25% methanol in PBS for 10 min each at room temperature. For embryos with tails covering part of heart surface, tails were removed before staining. Embryos were blocked in PBS-containing 0.1% Triton X-100 and 5% normal donkey serum for 1 h at 4 °C. Samples were then incubated in block solution containing 50% ESR antibody (Abcam, ab27595) overnight at 4 °C, followed by five times wash in PBS-containing 5% normal donkey serum. Samples were then incubated with secondary antibody anti-rabbit conjugated with peroxidase (Vector lab, 1:500) in blocking solution for 10 h, followed by five times PBS-containing 5% normal donkey serum wash. Finally, embryos were developed by chromogen DAB (Vector Lab) at room temperature to the desired extent. Images were taken under Leica stereomicroscope (M165 FC).

### Echocardiography

Echocardiographic measurements were performed on adult mice, which were anesthetized with isofluorane via a digital ultrasound system (Vevo 2100 Imaging System, Visual Sonics). Heart rate and LV dimensions, including diastolic and systolic wall thicknesses and LV end-diastolic and end-systolic chamber dimensions, were measured from two-dimentional short-axis views under M-mode tracings at the level of the papillary muscle. LV mass and functional parameters, such as percentage of fractional shortening (FS) and ejection fraction (EF), were calculated using the above original parameters and accompanying software.

### Statistics

Data for two groups were analyzed by an unpaired student’s *t* test, whereas comparison between more than two groups was performed using an ANOVA followed by Tukey’s multiple comparison test. Significance was accepted when *P < *0.05. All data are presented as mean ± SEM.

### Data availability

Data supporting the findings of this study are available within the article and its Supplementary Information files, and from the corresponding author upon reasonable request.

## Electronic supplementary material

Supplementary Information
