# Supplementary material for: Identification of a hybrid myocardial zone in the mammalian heart after birth
Source: Nat Commun. 2017 Jul 20;8:87. doi: 10.1038/s41467-017-00118-1 (PMC5519540; doi:10.1038/s41467-017-00118-1)
Supplement: Supplementary file 1 — Supplementary Information [file 41467_2017_118_MOESM1_ESM.pdf]

File Name: Supplementary Information
Description: Supplementary Figures and Supplementary Table.

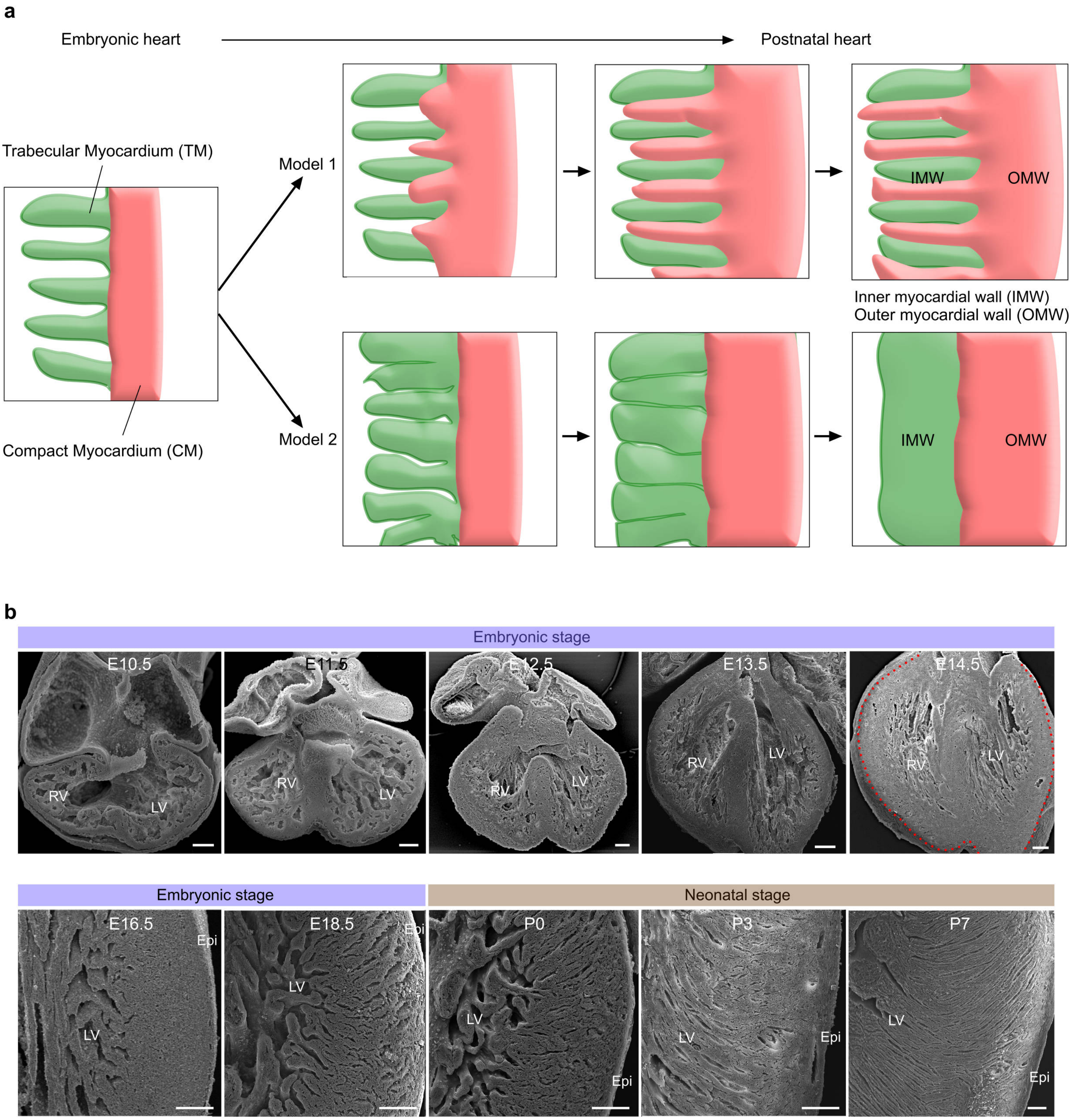

Supplementary Figure 1 Trabecular layer is transformed into compact myocardium after birth. (**a**) Schematic figure showing trabecular compaction models. Model 1 indicates expansion of cardiomyocytes from fetal compact myocardium into intertrabecular spaces to form inner myocardial wall (IMW) of postnatal ventricular wall. Model 2 indicates coalescence of fetal trabecular myocardium into IMW of postnatal ventricular wall. These simplified schematics do not fully capture the 3D geometry of direction. (**b**) Scanning electronic microscopy of embryonic hearts (E10.5, E11.5, E12.5, E13.5, E14.5, E16.5 and E18.5) and neonatal hearts (P0, P3 and P7). Trabecular coalescence is initiated at late embryonic and neonatal stages. Red dotted line indicates epicardium. Epi, epicardium; RV, right ventricle; LV, left ventricle. Scale bars, 100 um.

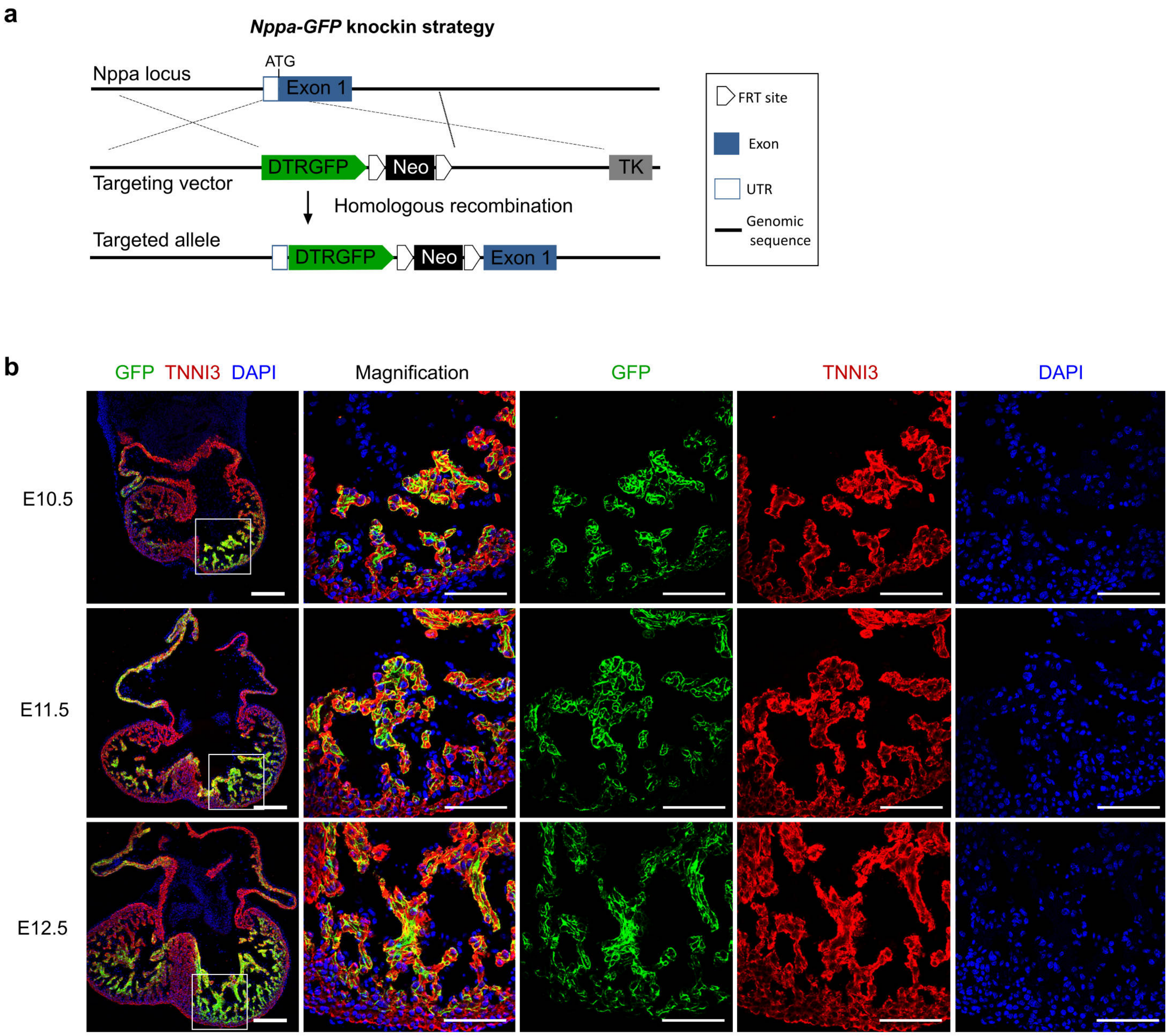

Supplementary Figure 2 Generation and characterization of Nppa-GFP mouse line. **(a)** Strategy of *Nppa-GFP* mouse generation by homologous recombination. **(b)** Immunostaining for GFP and TNNI3 on heart sections of E10.5 - E12.5 embryos. Scale bars, 100 µm.

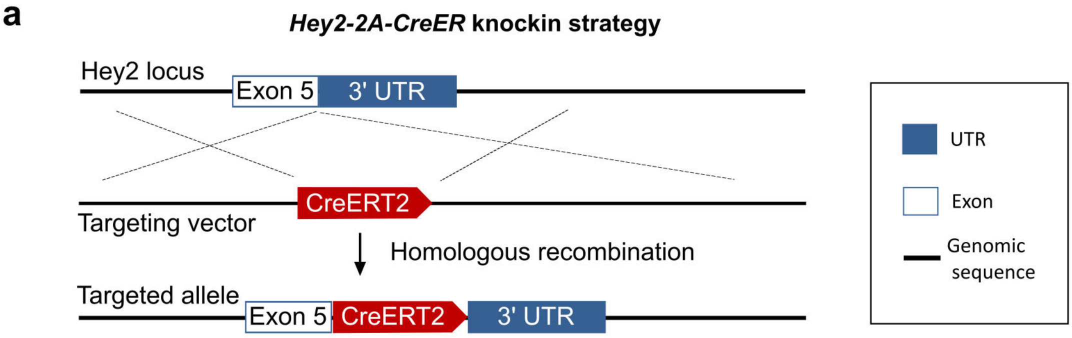

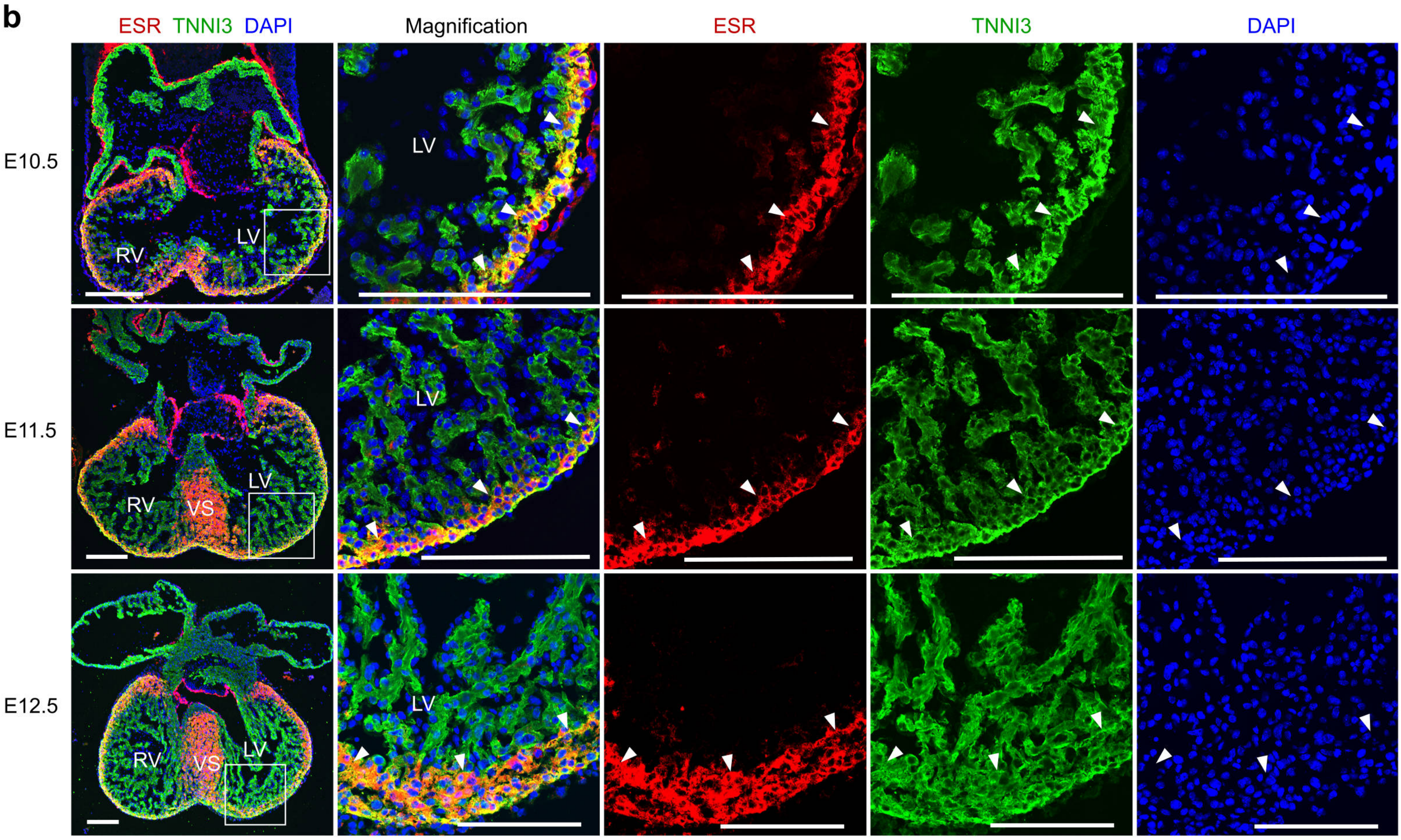

Supplementary Figure 3 Generation and characterization of *Hey2-2A-CreER* mouse line. **(a)** Schematic showing strategy for knock-in of 2A-CreER into Hey2 translational stop before 3' UTR by homologous recombination. **(b)** Immunostaining of ESR and TNNI3 showed that Hey2 expression is restricted to compact myocardium (arrowheads). RV, right ventricle; VS, ventricular septum; LV, left ventricle. Scale bars, 200 μm.

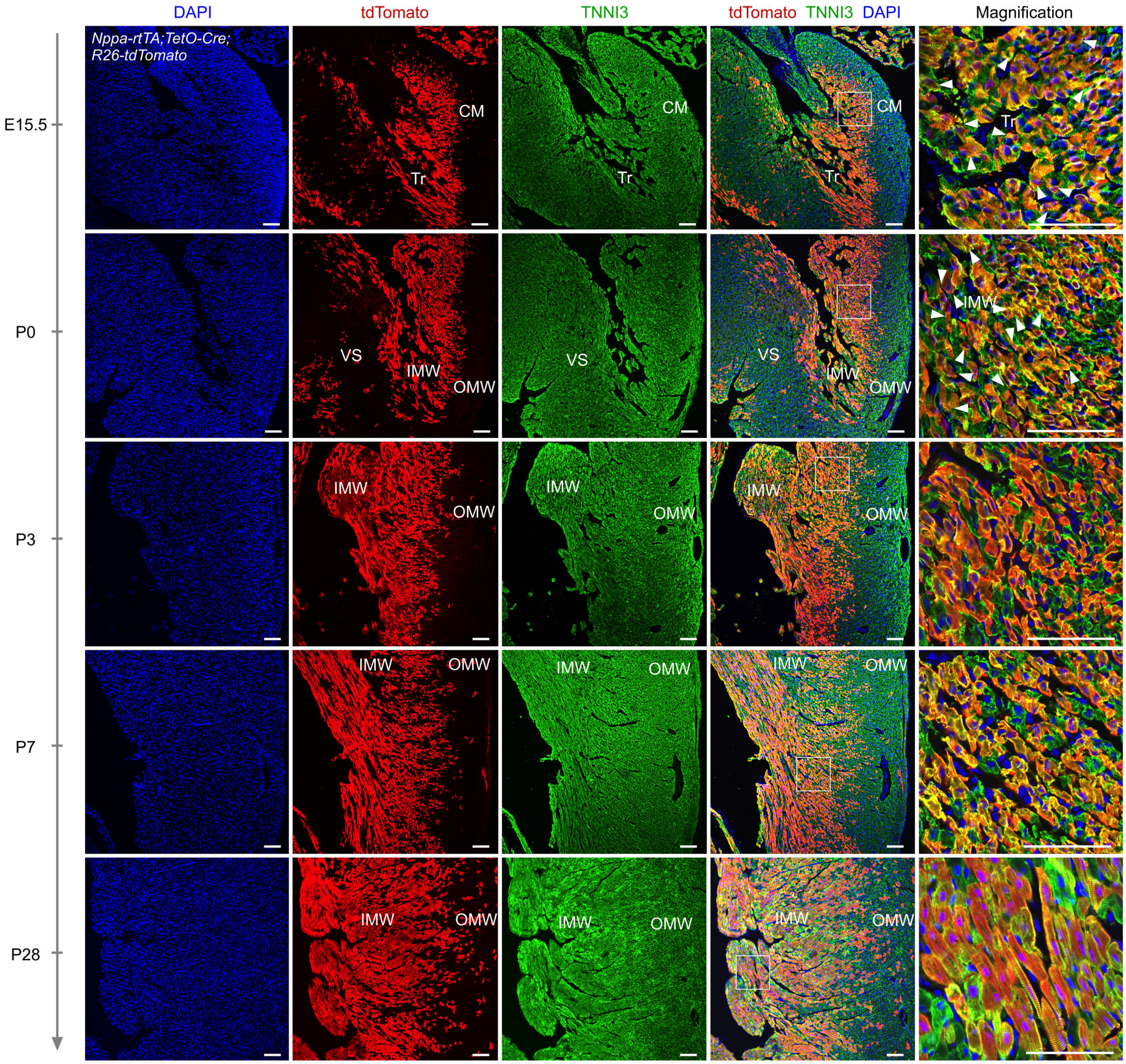

Supplementary Figure 4 Trabecular myocardium forms the inner myocardial wall in postnatal stage. Immunostaining for tdTomato and TNNI3 on E15.5, P0, P3, P7 and P28 *Nppa-rtTA;TetO-Cre;R26-tdTomato* hearts. Tr, trabecular layer; CM, compact myocardium; IMW, inner myocardial wall; MMW, middle myocardial wall; OMW, outer myocardial wall. Scale bars, 100 μm.

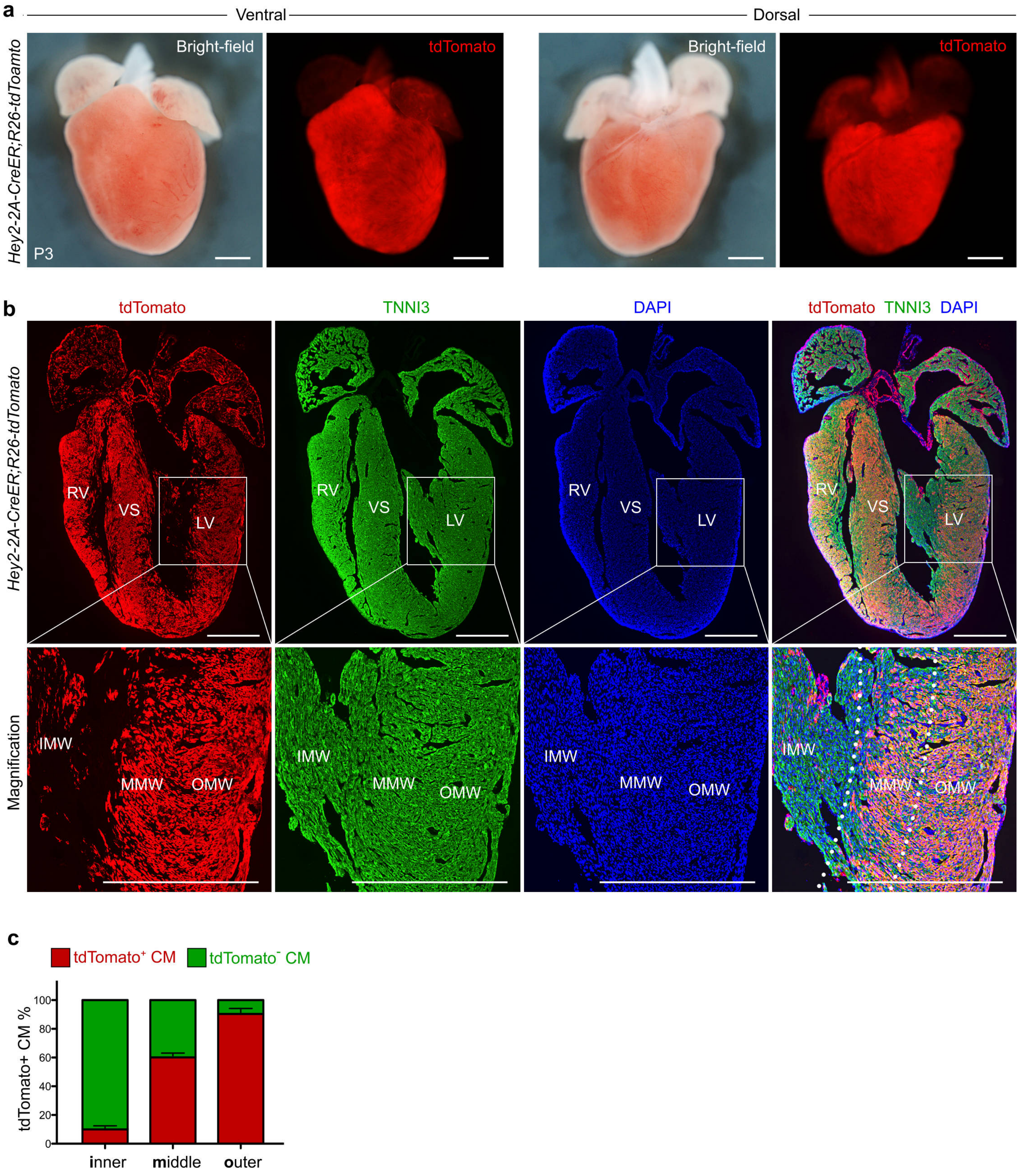

Supplementary Figure 5 Fate mapping of Hey2-derived cells in postnatal heart. **(a)** Whole-mount bright-field and fluorescence view of postnatal day 3 (P3) heart from *Hey2-2A-CreER;R26-tdTomato* mice. Tamoxifen was induced at E12.5. **(b)** Immunostaining for tdTomato and TNNI3 on heart sections shows most cardiomyocytes were not labeled in the inner myocardial wall (IMW), including papillary muscle. **(c)** Quantification of the tdTomato+ cardiomyocytes (CM) in the inner, middle and outer myocardial wall of P3 hearts. n = 4. Scale bars, 0.5 mm.

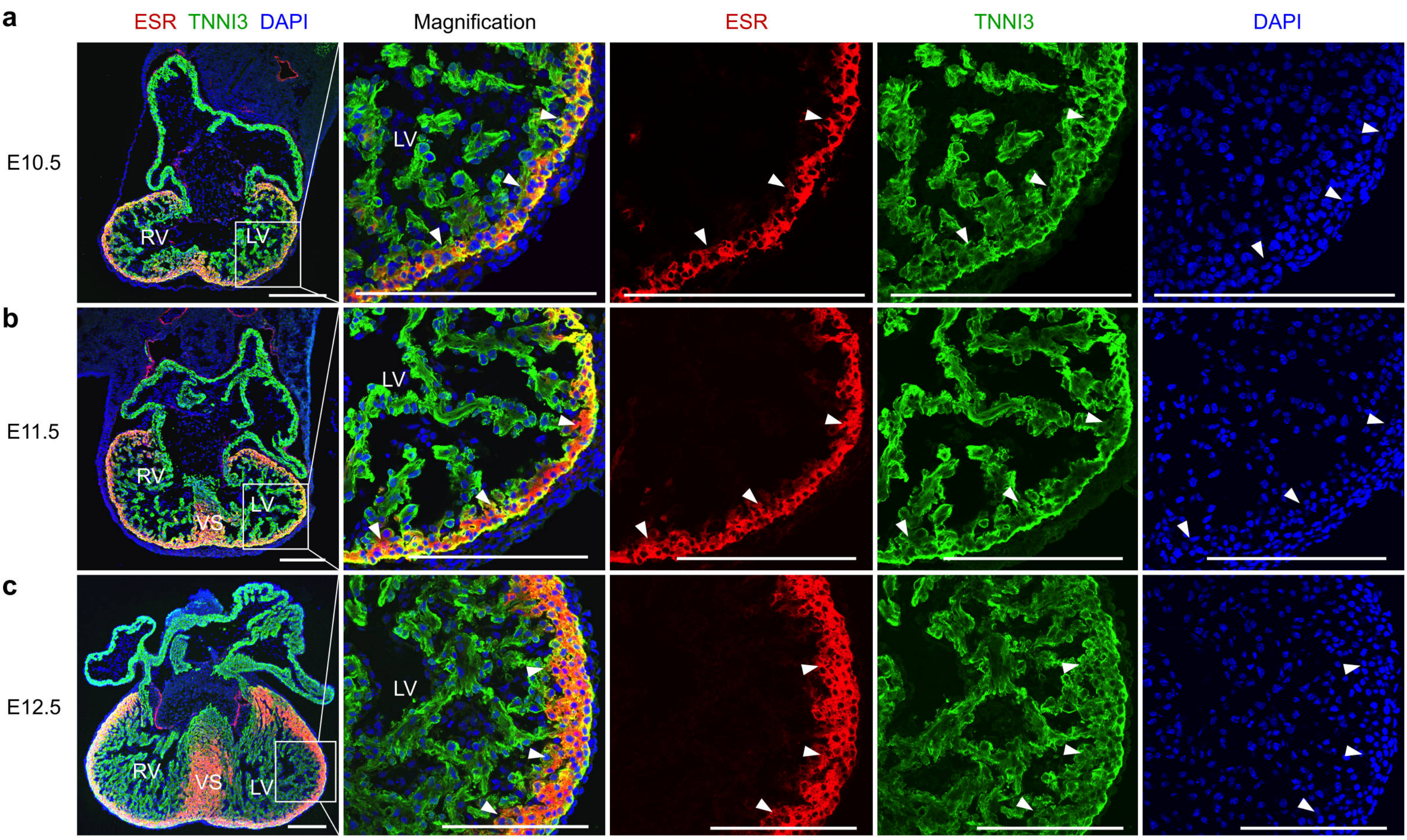

Supplementary Figure 6 Expression of CreER in *Hey2-CreER* mouse heart. **(a-c)** Immunostaining of ESR and TNNI3 showed that Hey2 expression is enriched in compact myocardium (arrowheads). RV, right ventricle; VS, ventricular septum; LV, left ventricle. Scale bars, 200 μm.

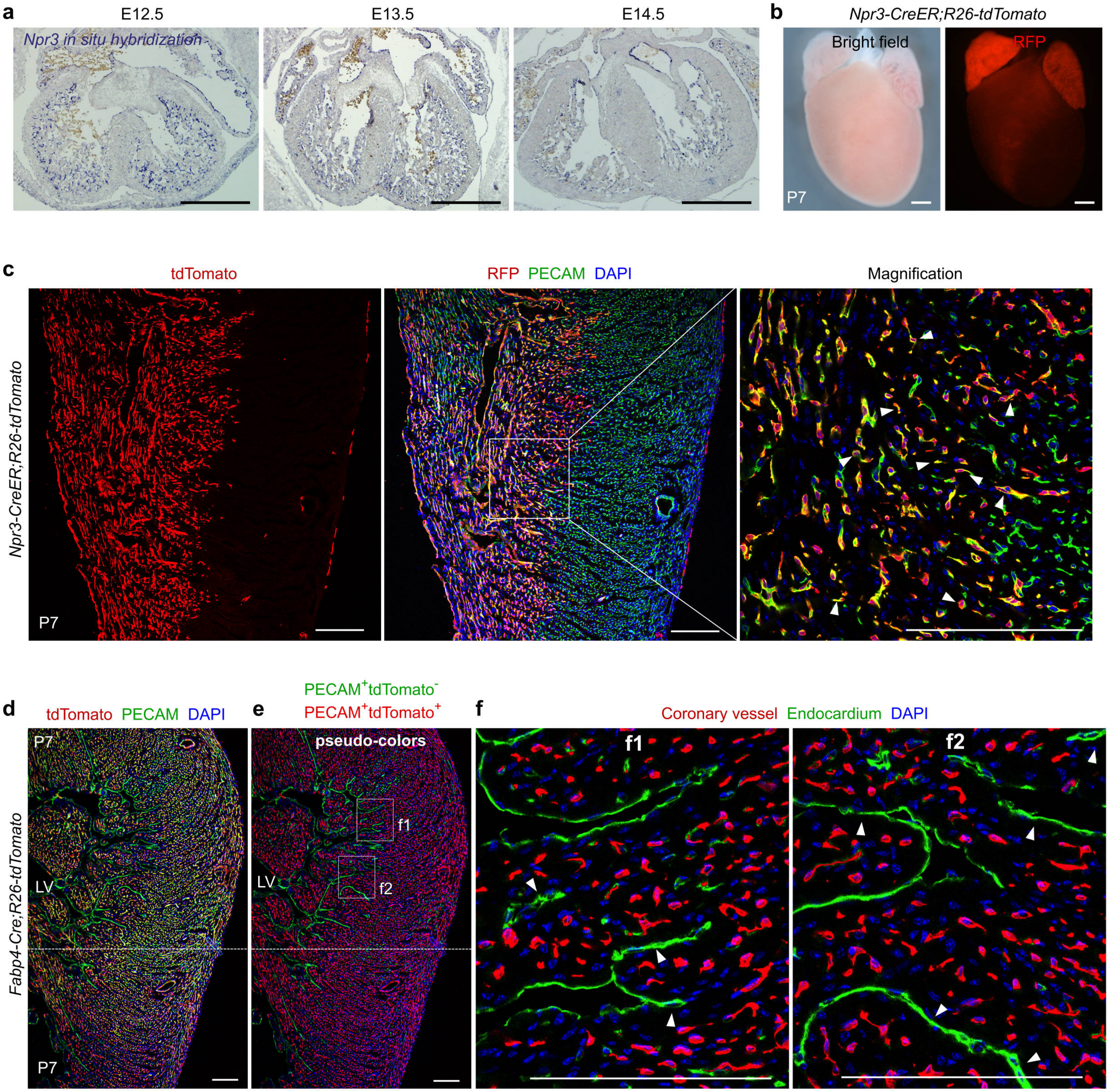

Supplementary Figure 7 Endocardium-derived cells are trapped in the inner wall of neonatal heart. **(a)** Images showing *in situ* hybridization of Npr3 on E12.5 - E14.5 heart sections. **(b)** Whole-mount bright-field and fluorescence view of P7 *Npr3-CreER;R26-tdTomato* heart. Tamoxifen was administered at E13.5. **(c)** Immunostaining for tdTomato and PECAM showing endocardium-derived coronary vessels (arrowheads) in the inner myocardial wall of neonatal heart. **(d)** Immunostaining for tdTomato and PECAM on sections of P7 *Fabp4-Cre;R26-tdTomato* mouse heart. Dotted line indicates the border of two merged images. **(e)** For contrast, pictures in d was pseudo-colored by green fluorescence for PECAM+tdTomato- cells and red fluorescence for PECAM+tdTomato+ cells. **(f)** Magnified images showing a subset of remaining endocardial cells (arrowheads) lining in-between blocks of myocardium in the inner ventricle wall. Scale bars, 500 μm in a,b; 200 μm in c,d. LV, left ventricle.

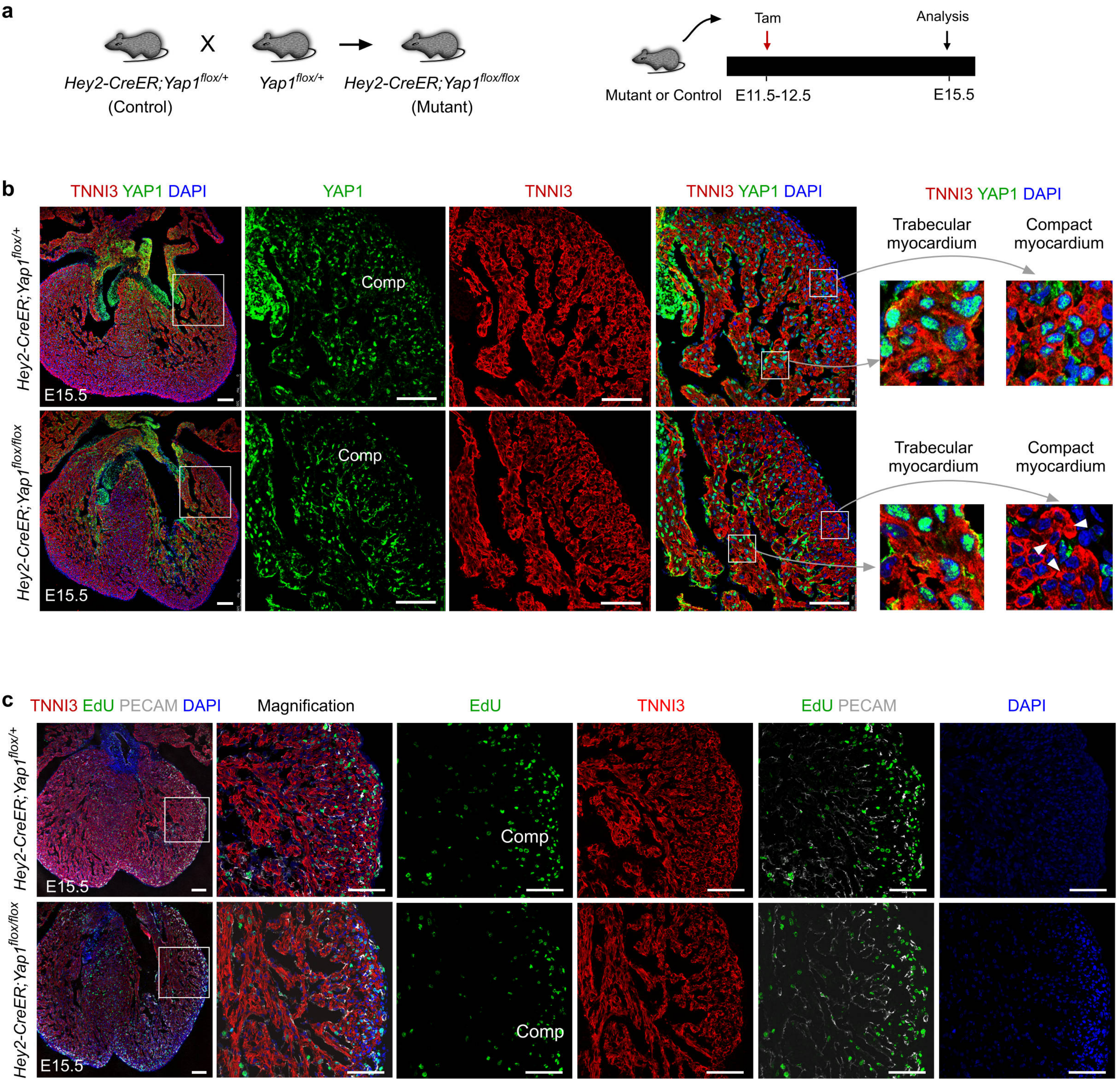

Supplementary Figure 8 Deletion of Yap1 by *Hey2-CreER* lead to reduced proliferation of cardiomyocytes in compact myocardium. **(a)** Schematic figure showing experimental strategy. **(b)** Immunostaining for TNNI3 and YAP1 on E15.5 control and mutant heart sections. YAP1 was detected in cardiomyocytes of trabecular but not compact myocardium in mutant. Arrowheads indicate YAP1^{-} cardiomyocytes in compact myocardium of mutant heart. **(c)** Immunostaining for TNNI3, EdU and PECAM on E15.5 control and mutant heart sections. Scale bars, 100 μm.

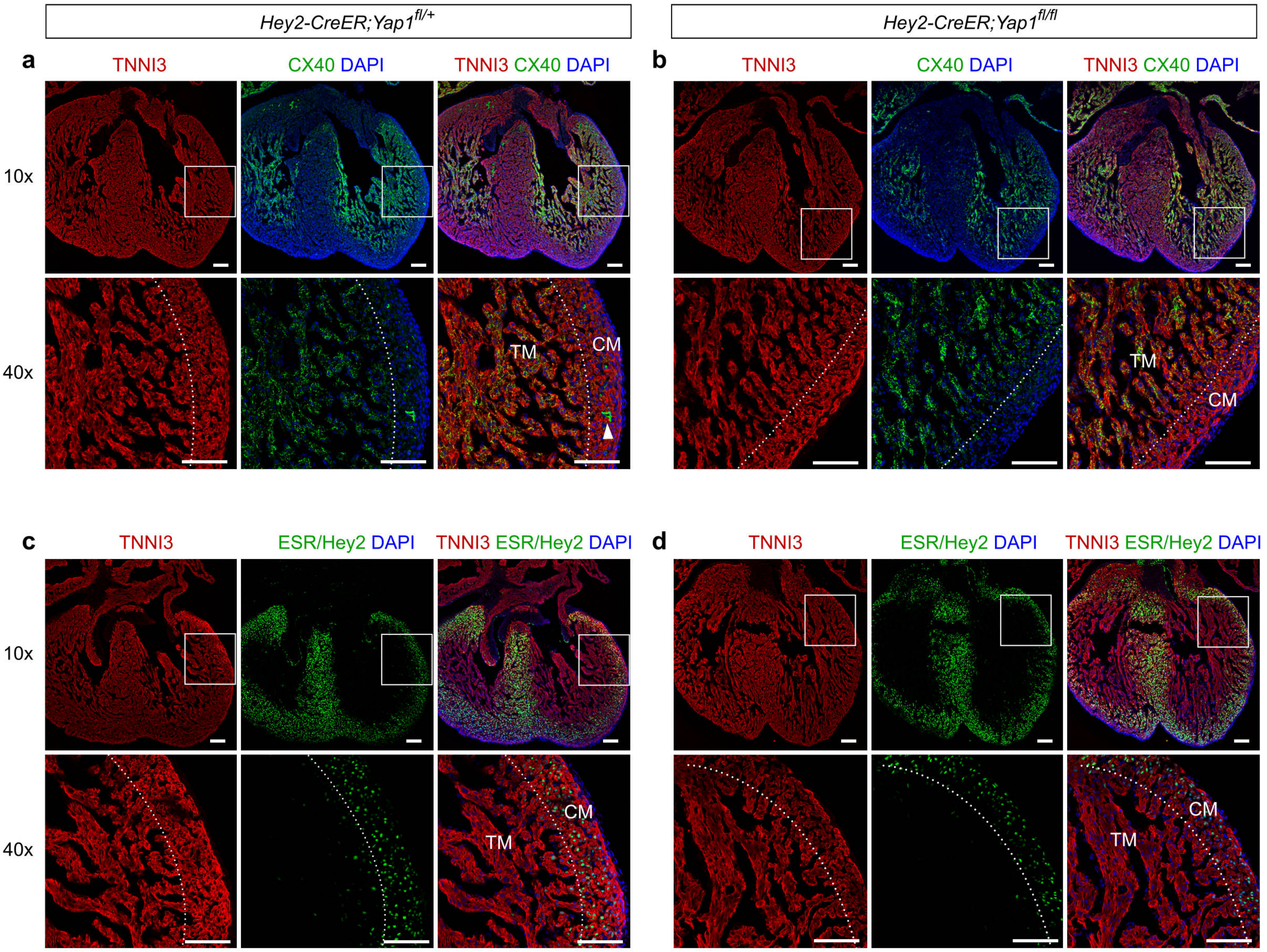

Supplementary Figure 9 Expression of compact and trabecular myocardial markers in YAP1 knockout heart. **(a,b)** Immunostaining for TNNI3 and CX40 on the control and mutant heart sections. Arrowhead indicates CX40+ artery in the compact myocardium. Dotted line demarcates the border between trabecular myocardium (TM) and compact myocardium (CM). **(c,d)** Immunostaining for TNNI3 and ESR, as surrogate for Hey2, on heart sections. Dotted line demarcates the border between trabecular myocardium (TM) and compact myocardium (CM). Scale bars, 100 μm.

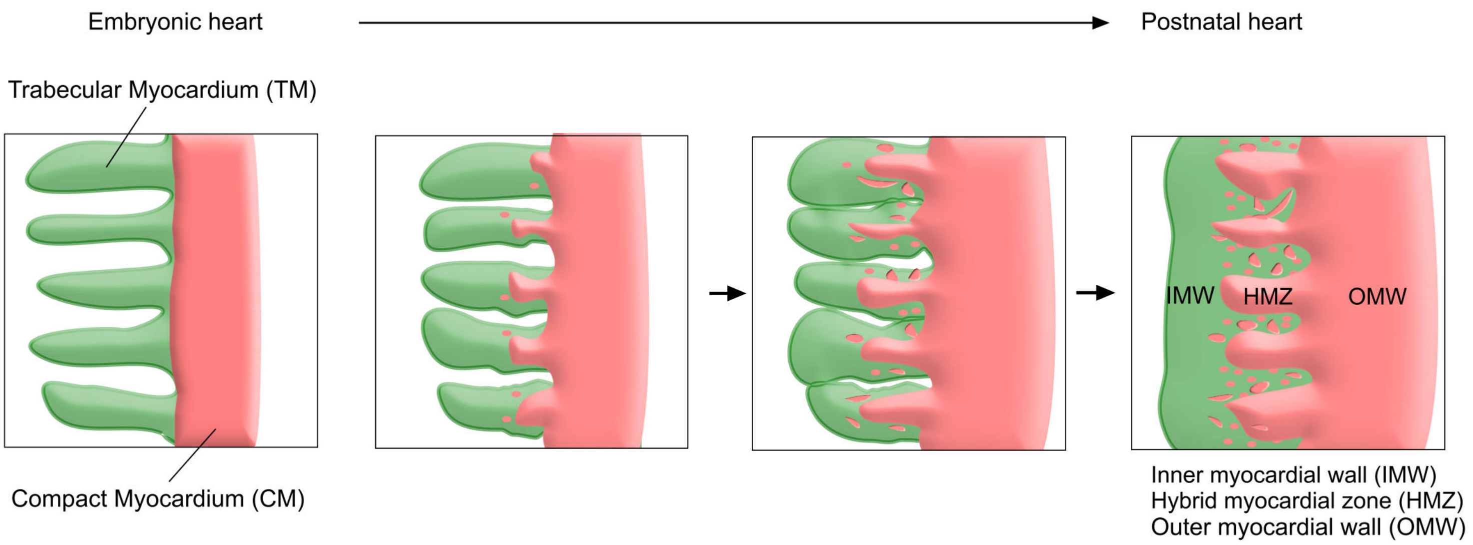

Supplementary Figure 10 Cartoon image showing the model proposed by this study. The compact myocardium in embryonic heart expand in epicardial-to-endocardial direction, and increase the volume of trabecular myocardium, which subsequently compress the intertrabecular spaces and facilitate trabecular coalescence. Compact myocardium does not contribute significantly to the innermost myocardial wall. These simplified schematics do not fully capture the 3D geometry of direction.

Supplementary Table 1. Primer sequences used in *in situ* hybridization

Gene	Forward	Reverse
Nppa	TTCTCCATCACCCTGGGCTTCTTC	AAGCTGTTGCAGCCTAGTCCAC
Hey2	CCAGAGGCTCATTGACACCAAC	TTATCGCTTTCTCCACACAGCAG
Npr3	TGGAGGAGAGGAGACAAACACGAC	GGACAGAGGACAGCAGAGAAAACG
